# Multi-focal control of mitochondrial gene expression by oncogenic MYC provides potential therapeutic targets in cancer

**DOI:** 10.18632/oncotarget.11718

**Published:** 2016-08-31

**Authors:** Amanda R. Oran, Clare M. Adams, Xiao-yong Zhang, Victoria J. Gennaro, Harla K. Pfeiffer, Hestia S. Mellert, Hans E. Seidel, Kirsten Mascioli, Jordan Kaplan, Mahmoud R. Gaballa, Chen Shen, Isidore Rigoutsos, Michael P. King, Justin L. Cotney, Jamie J. Arnold, Suresh D. Sharma, Ubaldo E. Martinez, Christopher R. Vakoc, Lewis A. Chodosh, James E. Thompson, James E. Bradner, Craig E. Cameron, Gerald S. Shadel, Christine M. Eischen, Steven B. McMahon

**Affiliations:** ^1^ Department of Cancer Biology, Thomas Jefferson University, Philadelphia, PA, USA; ^2^ Biomedical Graduate Studies, University of Pennsylvania, Philadelphia, PA, USA; ^3^ Department of Cancer Biology and Abramson Family Cancer Research Institute, University of Pennsylvania School of Medicine, Philadelphia, PA, USA; ^4^ Cold Spring Harbor Laboratory, Cold Spring Harbor, NY, USA; ^5^ Molecular and Cellular Biology Program, Stony Brook University, Stony Brook, NY, USA; ^6^ Department of Biochemistry, Thomas Jefferson University, Philadelphia, PA, USA; ^7^ Genetics and Genome Sciences, University of Connecticut Health, Farmington, CT, USA; ^8^ Department of Biochemistry & Molecular Biology, The Pennsylvania State University, University Park, PA, USA; ^9^ Leukemia Service, Department of Medicine, Roswell Park Cancer Institute, Buffalo, NY, USA; ^10^ Department of Medical Oncology, Dana-Farber Cancer Institute, Harvard Medical School, Boston, MA, USA; ^11^ Department of Pathology, Yale School of Medicine, New Haven, CT, USA; ^12^ Department of Genetics, Yale School of Medicine, New Haven, CT, USA

**Keywords:** MYC, mitochondria, mitochondrial gene expression, tigecycline, synthetic lethality

## Abstract

Despite ubiquitous activation in human cancer, essential downstream effector pathways of the MYC transcription factor have been difficult to define and target. Using a structure/function-based approach, we identified the mitochondrial RNA polymerase (POLRMT) locus as a critical downstream target of MYC. The multifunctional POLRMT enzyme controls mitochondrial gene expression, a process required both for mitochondrial function and mitochondrial biogenesis. We further demonstrate that inhibition of this newly defined MYC effector pathway causes robust and selective tumor cell apoptosis, *via* an acute, checkpoint-like mechanism linked to aberrant electron transport chain complex assembly and mitochondrial reactive oxygen species (ROS) production. Fortuitously, MYC-dependent tumor cell death can be induced by inhibiting the mitochondrial gene expression pathway using a variety of strategies, including treatment with FDA-approved antibiotics. *In vivo* studies using a mouse model of Burkitt's Lymphoma provide pre-clinical evidence that these antibiotics can successfully block progression of MYC-dependent tumors.

## INTRODUCTION

Overexpression of the c-*MYC* oncogene occurs in most human cancers [1]. The MYC transcription factor regulates expression of several thousand genes in order to drive cell cycle progression and malignant transformation [2, 3]. Among this array of MYC targets, it remains unclear which genes play an essential role in malignant transformation. The identification of this essential subset of MYC targets is important for two main reasons. First, they may provide an understanding of which cellular pathways and processes MYC regulates to reprogram cellular physiology. Second, these targets may serve as points of therapeutic intervention in the face of our historic inability to target MYC directly. To address this knowledge gap, we previously performed a structure-function based screen to identify MYC targets linked to malignant transformation [4]. This screen identified a subset of ~20 MYC targets as specifically correlated with transformation [4]. Of this small number of genes, subsequent empirical analysis has confirmed that several play critical roles in the MYC pathway [5, 6]. Among the genes identified by this screen was that encoding the mitochondrial RNA polymerase (POLRMT), also known as mtRNAP.

POLRMT encodes the rate-limiting RNA polymerase enzyme which controls transcription of the small, circular mitochondrial genome [7]. POLRMT also contributes to mitochondrial gene expression by regulating mtDNA replication *via* generation of RNA primers [8], and interacting with the mitochondrial rRNA methyltransferase TFB1M to mediate proper assembly of the small mitochondrial ribosome subunit [9].

The mitochondrial proteome is encoded by distinct nuclear and mitochondrial genomes. It is well-established that MYC drives the nuclear-encoded portion of the mitochondrial biogenesis program [10, 11]. The discovery of POLRMT as an essential target of MYC provides a mechanistic explanation for how MYC also regulates mitochondrial genome transcription in order to fully reprogram mitochondrial function.

Transport of electrons is one of the essential processes within mitochondria and occurs *via* the five complexes of the electron transport chain (ETC). Four of these complexes consist of subunits encoded by both nuclear- and mitochondrial-encoded genes. Remarkably, interference with MYC's ability to induce mitochondrial transcription results in an imbalance in electron transport chain complex components, induction of mitochondrial ROS and ultimately converts the cellular response to MYC activation from cell proliferation to apoptosis. These effects are specific to tumor cells harboring MYC activation and can be exploited *in vivo* using tetracycline family antibiotics to block mitochondrial translation. Inhibition of this pathway fully eradicated tumor formation in the Eμ-*myc* transgenic model of human Burkitt's Lymphoma.

## RESULTS

### MYC controls transcription of the gene encoding the mitochondrial RNA polymerase POLRMT

**Figure 1:**
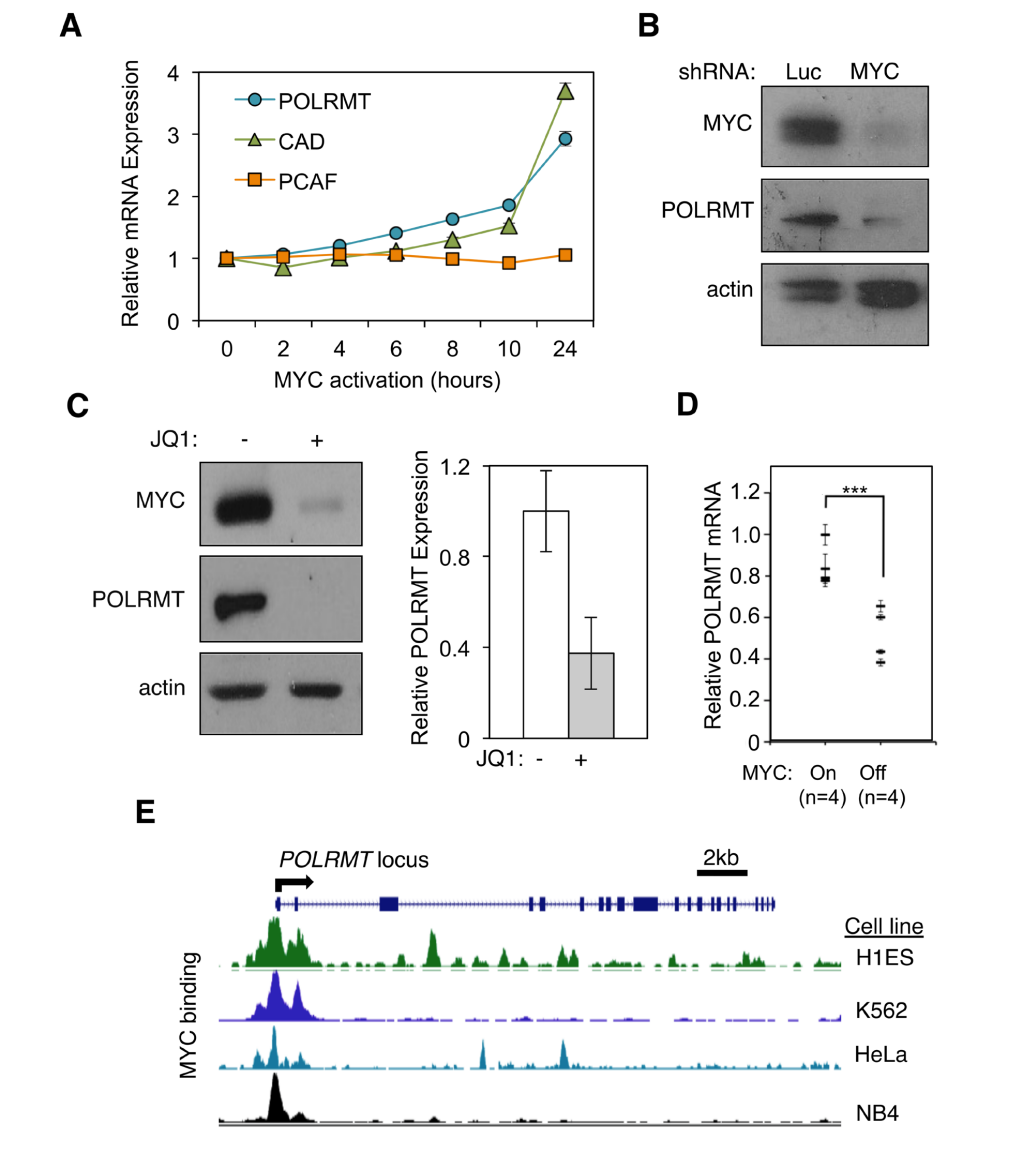
POLRMT is a direct transcriptional target of the MYC oncoprotein **A.** 2091 MYC/ER cells were treated with 4-hydroxytamoxifen (4-OHT) to induce MYC activity. Cells were harvested at the indicated time points and analyzed by quantitative real-time PCR (qRT-PCR) to show the kinetics of gene induction. Error bars represent SD, *n* = 3. **B.** H1299 cells were infected with lentiviral MYC shRNA or Luciferase (Luc) shRNA as a control. Cells were harvested and whole cells lysates were analyzed by Western blot for the indicated proteins. **C.** Raji cells were treated with JQ1 to deplete MYC levels. Cells were harvested and whole cell lysates were analyzed by Western blot (left) for the indicated proteins and qRT-PCR (right) for POLRMT expression. Error bars represent SD, *n* = 3. **D.** Mammary tumors were induced in six week old female bitransgenic MMTV-rtTA;TetO-MYC mice *via* administration of 2 mg/mL doxycycline in drinking water. After mammary tumor formation, doxycycline was withdrawn from the water of 4 mice (MYC OFF) while the other mice were maintained on doxycycline (MYC ON). 96 hours after doxycycline withdrawal, tumors were harvested and mRNA analyzed by qRT-PCR. Error bars represent SD, *n* = 4. ***p < .001 Student's *t*-test (two-sided). **E.** Modified image taken from UCSC genome browser (Assembly GRCh37/hg19) ENCODE transcription factor ChIP track showing MYC binding at the *POLRMT* locus in a variety of cell lines (http://genome.ucsc.edu).

The oncogenic transcription factor MYC binds to several thousand loci in the human genome [12]. However, it remains unclear which of these MYC targets are essential for MYC function (reviewed in [13]). To refine our understanding of MYC targets specifically relevant to malignant transformation, we conducted an expression profiling screen designed to identify downstream targets whose transcriptional activation correlates with cellular transformation [4]. We report here that the nuclear-encoded mitochondrial RNA polymerase gene POLRMT is a direct MYC target. In brief, primary human fibroblast cells were engineered to express oncogenic levels of MYC *via* a conditional allele in which MYC is fused to a mutated portion of the estrogen receptor (ER). This fusion protein allows for induction of oncogenic MYC activity upon treatment with the estrogen analog 4-hydroxytamoxifen (4-OHT) [14]. As validation, the kinetics and absolute level of POLRMT induction upon 4-OHT treatment were found to be similar to those of the well-characterized MYC target gene CAD, whereas the non-target gene PCAF was unaffected by MYC activation. (Figure 1A). Supporting this, the study which first demonstrated the role of MYC in mitochondrial function identified POLRMT as one of 281 mitochondrial ontology genes among a total of 2679 MYC responsive genes [11].

Levels of ectopic MYC used in Figure 1A mimic those in human tumors where MYC is amplified or translocated. To more rigorously assess the physiological relevance of POLRMT induction, we examined whether endogenous MYC also regulates POLRMT transcription. For this purpose, endogenous MYC was depleted from a panel of cell lines from different lineages using both genetic and biochemical methods. In order to assess POLRMT expression in a variety of cell lines exhibiting endogenous MYC overexpression, the human Burkitt's lymphoma cell line Raji was used in addition to the epithelial line H1299 to represent a lymphoid model with MYC translocation. In the non-small cell lung cancer cell line H1299, depletion of MYC *via* shRNA led to a decrease in POLRMT protein (Figure 1B). As an alternative method of depleting endogenous MYC levels, cells were treated with the BET bromodomain inhibitor JQ1, which inhibits MYC protein and transcript levels [15]. In Raji cells, treatment with JQ1 led to a decrease in POLRMT mRNA and protein, concomitant with decreased MYC (Figure 1C). Additionally, JQ1 treatment of the primary human fibroblast line LG1 and the acute myeloid leukemia cell line NOMO-1, the cell lines used to originally characterize the effect of JQ1 on MYC expression, led to decreased levels of POLRMT mRNA (Supplemental Figure S1A). Considered together, these data suggest that MYC is an essential regulator of the mitochondrial RNA polymerase POLRMT in a broad array of tumor cell lineages.

To assess whether the findings linking MYC and POLRMT expression extend to an *in vivo* setting, POLRMT mRNA expression was quantified in tumors from a mouse model of MYC-driven mammary cancer. In these animals, MYC expression is mammary gland specific and doxycycline-inducible [16]. Tumors were induced in 6-week old mice and after appearance of palpable mammary tumors, a subset of mice were withdrawn from doxycycline to inhibit MYC expression. Analysis of mRNA levels from harvested tumors demonstrated that POLRMT transcript levels tightly correlate with MYC activity *in vivo*, with a significant decrease in POLRMT mRNA observed in mice when MYC activity was reduced even briefly (Figure 1D). Furthermore, analysis of TCGA datasets using the cBioPortal for Cancer Genomics indicated a correlation between MYC amplification and/or increased mRNA expression and increased POLRMT mRNA expression in cancer patient samples. For example, datasets for glioblastoma and breast invasive carcinoma reveal a significant correlation between MYC and POLRMT, with *p* values of < 0.001 and .002, respectively [17, 18]. In addition, when analyzed using the Oncomine Cancer Profiling Database (www.oncomine.org), both MYC and POLRMT, as well as established MYC targets LDHA and BAG1, are overexpressed in Burkitt's lymphoma samples compared to normal B lymphocytes (Supplemental Figure S1B) [19]. Similarly, several cancer types including colorectal cancers, diffuse large B cell lymphoma, and multiple myeloma display increased POLRMT expression correlated with increased MYC expression in cancer cells compared to normal control cells (Supplemental Figure S1C) [20-22].

To address whether POLRMT represents a primary target of MYC, the binding of endogenous MYC to the *POLRMT* locus was evaluated. Within the genomic sequence of POLRMT, several matches to the consensus high-affinity binding site (E-box) for MYC/MAX dimers were identified. *In silico* analysis of the ENCODE transcription factor track in the UCSC genome browser showed MYC binding at multiple E-box sequences within the POLRMT locus in a variety of human cell types (Figure 1E) [23, 24]. The direct binding of MYC to the *POLRMT* locus is also supported by genome-wide studies in murine embryonic stem cells showing occupancy of this region 5′ of the POLRMT locus by MYC, using ChIP-seq [25]. We empirically confirmed specific recruitment of endogenous MYC to these sites by ChIP in the primary human fibroblast cell line 2091 (Supplemental Figure S1D).

### MYC controls mitochondrial transcription and mitochondrial genome content *via* POLRMT induction

**Figure 2:**
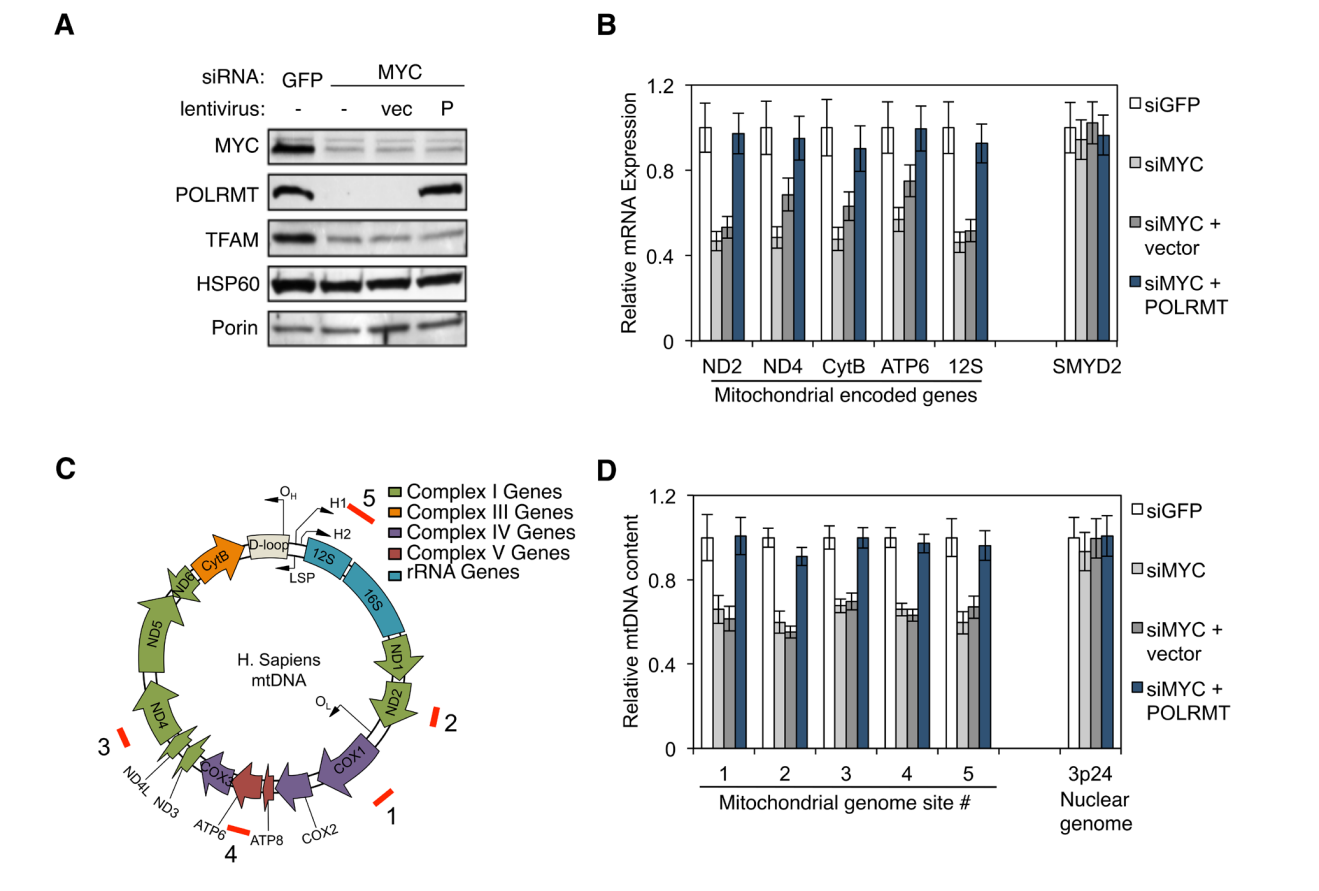
MYC regulates mitochondrial transcription and mtDNA replication *via* POLRMT **A.** H1299 cells were transfected with MYC siRNA or GFP siRNA as a control. 24 hours post-transfection, one group of MYC depleted cells was infected with lentivirus expressing human POLRMT (P) or the empty vector (vec). Three days post-transfection, cells were harvested and mitochondrial proteins POLRMT, TFAM, HSP60 and Porin were measured from purified mitochondria. The level of MYC depletion was evaluated from whole cell lysates. **B.** Cells described in (A) were analyzed by qRT-PCR for mitochondrial gene expression. Nuclear gene SMYD2 was used as a control. Error bars represent SD, *n* = 3. **C.** Map of the human mitochondrial genome indicating ORF, tRNA, rRNA and gene locations. Locations of the primer sets used for quantifying mtDNA genome in (D) are indicated (red bars labeled 1-5). **D.** Total cellular DNA from cells described in (A) was quantified by qPCR using primer sets shown in (C). Primers amplifying a region at 3p24 were used to validate the specificity of the mitochondrial/nuclear comparison. Error bars represent SD, *n* = 3.

As discussed, POLRMT catalyzes transcription of the 16.6kb mitochondrial genome. To determine whether MYC controls mitochondrial gene expression *via* POLRMT induction, expression of a panel of mitochondrial genes was assessed in H1299 cells. Western blot analysis of mitochondrial lysates from these cells confirmed that MYC depletion resulted in the loss of POLRMT (Figure 2A). MYC knockdown also resulted in a partial loss of the mitochondrial transcription factor TFAM, an established MYC target [11]. Decreases in POLRMT and TFAM were not a result of a general defect in the importation of nuclear-encoded proteins into the mitochondria, since HSP60 protein levels in the mitochondria were not affected by MYC depletion. In order to assess whether the MYC-dependent loss of POLRMT results in a defect in mitochondrial transcription, qRT-PCR was performed for the mitochondrial-encoded genes ND2, ND4, CytB, ATP6 and 12S, as well as the nuclear gene SMYD2 (Figure 2B). This analysis revealed that depletion of MYC caused a decrease in all five of the mitochondrial transcripts assessed. SMYD2, which is not a MYC target, was unaffected by MYC depletion. In MYC-depleted cells, restoring POLRMT levels by ectopic expression (Figure 2A) was sufficient to rescue the mitochondrial transcripts to normal levels (Figure 2B), confirming that POLRMT is the limiting factor in their expression.

MYC regulates mitochondrial biogenesis [11], a process that requires increased mitochondrial DNA (mtDNA) synthesis. In addition to its role in gene expression, POLRMT also primes mtDNA replication [26, 27], contributing to formation of the D loop structure at the major origin of DNA replication within the mitochondrial genome [28]. This raises the possibility that POLRMT induction might explain the role of MYC in mitochondrial genome replication. In support of this model, MYC-depleted H1299 cells displayed a significant decrease in mitochondrial genome content when assessed at five independent regions of the mitochondrial genome (Figure 2C and 2D). Upon expression of ectopic POLRMT, mitochondrial genome content was fully rescued. In contrast, nuclear DNA content was unaffected by MYC depletion or POLRMT expression, as quantified at the control locus 3p24.

### POLRMT inhibition is synthetically lethal with oncogenic levels of MYC

**Figure 3:**
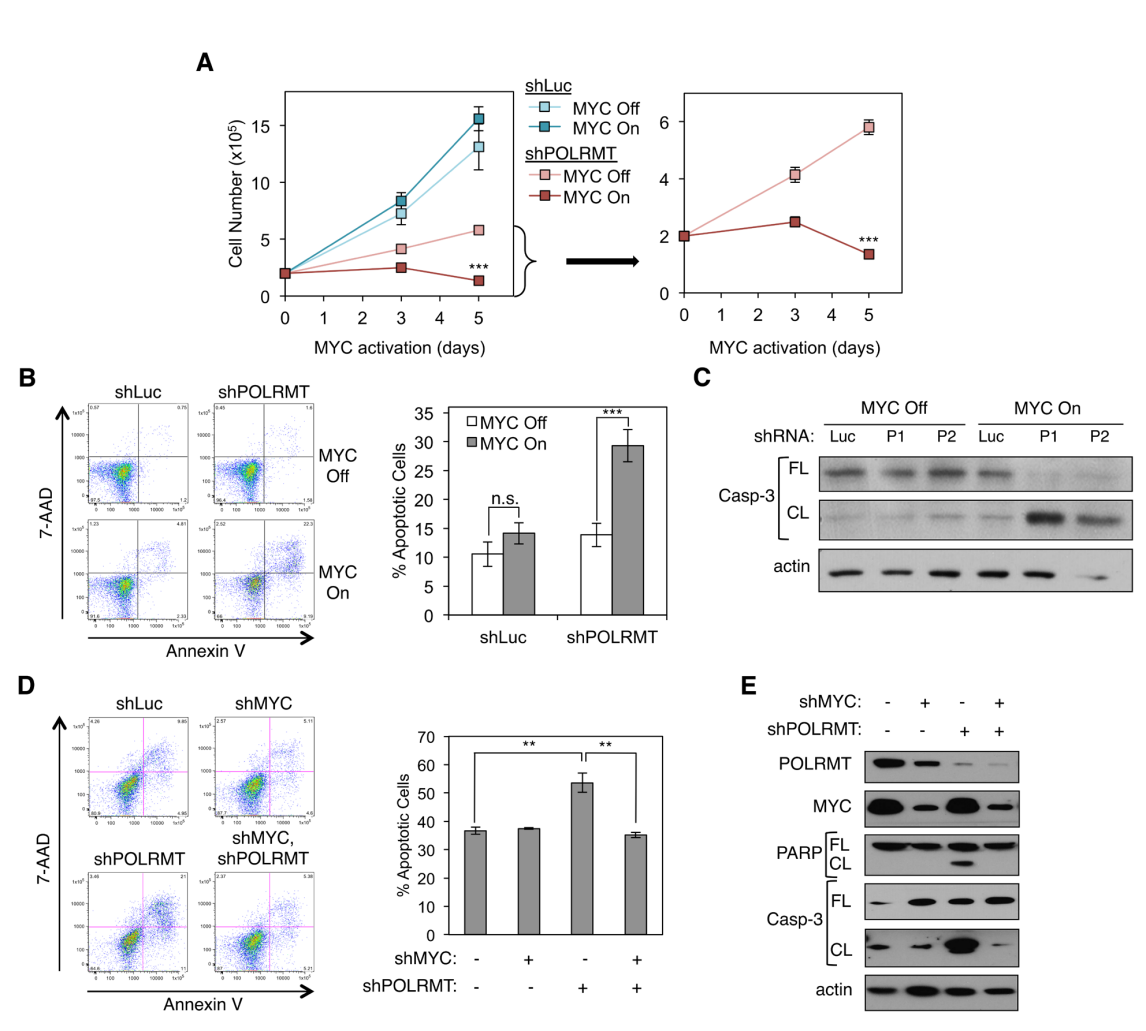
Synthetic Lethal Dependency of Deregulated MYC on POLRMT **A.** U2OS MYC/ER cells were infected with lentiviral POLRMT shRNA or Luciferase (Luc) shRNA as a control. Five days post-infection cells were seeded at equal densities and MYC activity was induced *via* treatment with 4-OHT (MYC On). Cells were counted on day three and day five post MYC-activation. shPOLRMT cell counts are shown with an adjusted scale (right). Error bars represent SD, *n* = 3. ***p < .001, Unpaired *t*-test. **B.** U2OS MYC/ER cells were depleted of POLRMT and treated with 4-OHT (MYC On), as in (A). Cells were harvested on day three post MYC-activation and stained with Annexin V and 7-AAD. A representative flow cytometry analysis (left) and quantification of the percent Annexin V positive cells (right) are shown. Error bars represent SEM, *n* = 18. ***p < .001; n.s., not significant, Unpaired *t*-test. **C.** U2OS MYC/ER cells were infected with two distinct lentiviral POLRMT shRNA plasmids (P1 and P2) or shLuc. Five days post-infection MYC was activated *via* treatment with 4-OHT (MYC On). Cells were harvested three days post MYC activation and whole cell lysates were analyzed by Western blot for the indicated proteins. **D.** HCT116 cells were infected with lentiviral POLRMT shRNA and/or MYC shRNA, or Luciferase shRNA. Five days post-infection cells were harvested and stained with Annexin V and 7-AAD. A representative flow cytometry analysis (left) and quantification of percent of Annexin V positive cells (right) is shown. Error bars represent SEM, *n* = 3. **p < .01; n.s., not significant, Unpaired *t*-test. **E.** Whole cell lysates of cells described in (D) were analyzed by Western blot for the indicated proteins. Casp-3, caspase-3; FL, full length; CL, cleaved.

As described earlier, POLRMT was identified in a screen for MYC targets correlated with malignant transformation [4]. To assess the functional requirement for POLRMT as a downstream MYC effector, the extent to which MYC-overexpressing cells require POLRMT was tested. A human osteosarcoma line U2OS expressing a conditional MYC/ER allele was used for this analysis, as these cells display robust MYC-dependent changes in either cell proliferation or apoptosis, depending on cellular contexts [29]. POLRMT was depleted from these cells using shRNA and cells were plated at equal numbers prior to MYC activation *via* treatment with 4-OHT. Over the course of 5 days, cell numbers were quantified (Figure 3A). The cells used in this experiment proliferate rapidly independent of MYC/ER activity. As expected, activation of MYC resulted in a small increase in cell number. In contrast, in cells where POLRMT induction was blocked, activation of MYC resulted in a substantial decrease in cell number. Apoptosis assays (both Annexin V staining and Western blotting for caspase-3 cleavage) demonstrated that this decrease in cell number resulted from a significant increase in apoptosis under conditions of MYC activation and POLRMT depletion (Figures 3B, 3C and S2A). Depletion of POLRMT without MYC activation slowed proliferation, but did not significantly affect cell viability (Figure 3A and 3B). Thus, loss of POLRMT induction was synthetically lethal only with oncogenic levels of MYC activation. These observations were consistent over the course of multiple experiments, using multiple shRNA molecules to target POLRMT (Figures 3B and 3C). Though levels of apoptosis are less than 100%, a clonogenic assay was performed to assess the replicative potential of surviving cells. Consistent with the proliferation assay discussed above, the remaining cells under synthetic lethal conditions did not exhibit significant replicative potential (data not shown).

While the conditional MYC/ER allele has been used extensively to mimic oncogenic levels of MYC function [14, 30], it was also important to assess the relevance of POLRMT under more physiological conditions. For this purpose, HCT116 colon carcinoma cells, which express high levels of endogenous MYC (Supplemental Figure S2B), were depleted of POLRMT, MYC, or both POLRMT and MYC using shRNA. Levels of apoptosis were then analyzed (Figures 3D and 3E). Annexin V staining revealed that cells with low POLRMT expression and high endogenous MYC displayed increased apoptosis (Figure 3D). Furthermore, depletion of MYC in addition to depletion of POLRMT rescued cell viability to a normal level. Apoptosis was also observed upon POLRMT depletion, as assessed using caspase-3 cleavage and PARP cleavage as markers (Figure 3E). As with Annexin V staining, apoptosis measured by caspase-3 cleavage and PARP cleavage were reduced upon depletion of MYC. To further extend these findings in another cell line expressing high endogenous MYC, Raji Burkitt's lymphoma cells were depleted of POLRMT using shRNA. In these cells, MYC is expressed at very high levels due to chromosomal translocation [31]. Consistent with the observations made in HCT116 cells, a decrease in POLRMT expression in combination with oncogenic, endogenous MYC expression led to increased apoptosis in Raji cells, as evaluated by Annexin V staining (Supplemental Figures S2C and S2D). These data confirm that POLRMT depletion is synthetically lethal with oncogenic levels of MYC, in a variety of human cancer cell lineages.

### Synthetic lethality can be induced pharmacologically *via* treatment with 2′CMeA

**Figure 4:**
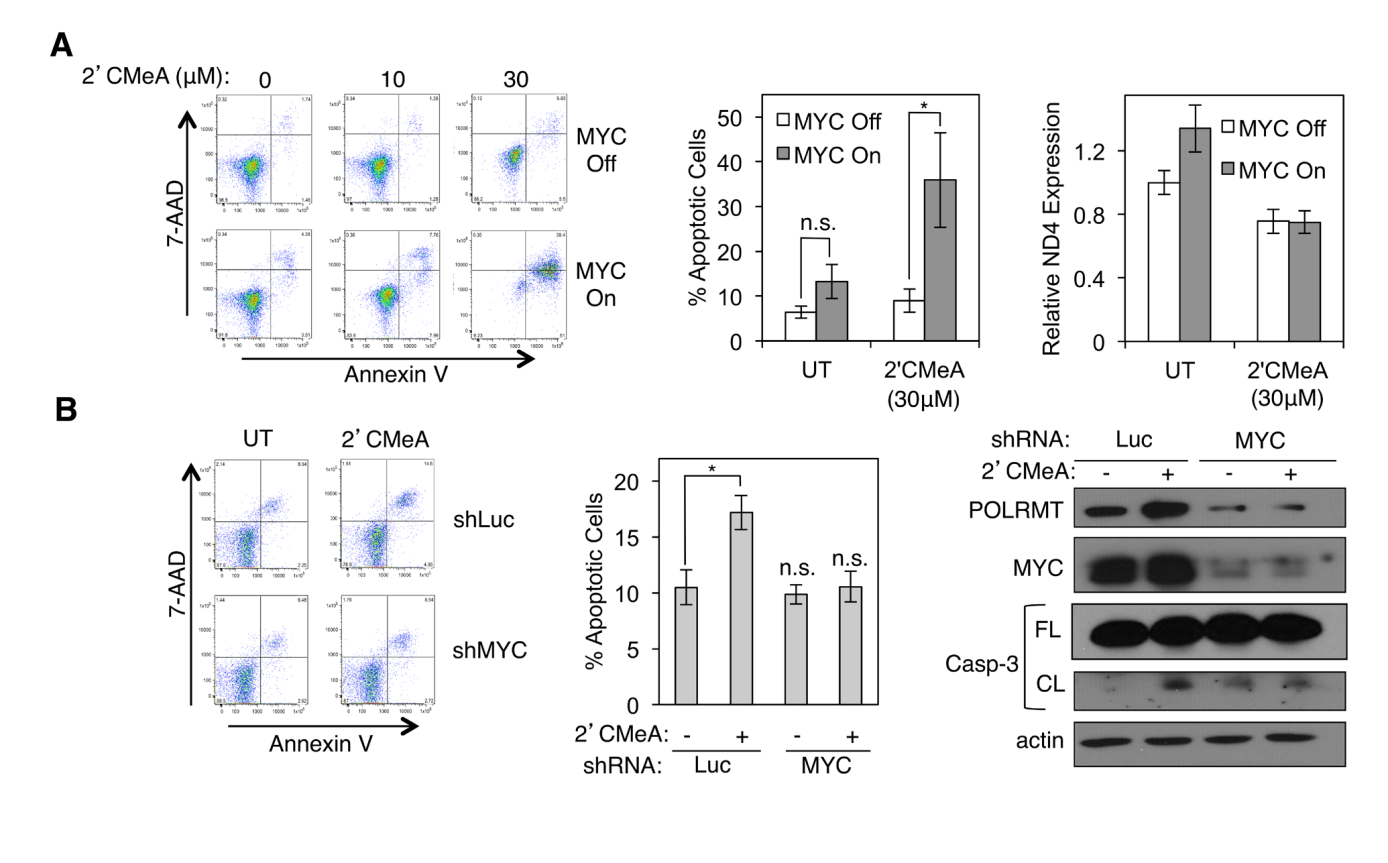
Synthetic lethality can be induced *via* treatment with 2′C Methyladenosine (2′CMeA) **A.** U2OS MYC/ER cells were treated with increasing concentrations of 2′CMeA, as indicated. MYC activity was induced *via* treatment with 4-OHT (MYC On). Three days post MYC-activation cells were harvested and stained with Annexin V and 7-AAD (left). Quantification of percent Annexin V positive cells at 30μM 2′CMeA dose is shown (middle). Expression of mitochondrial transcript ND4 was assessed by qRT-PCR (right). Error bars represent SEM (middle) and SD (right), *n* = 7. *p< .01; n.s. not significant, Unpaired *t*-test. **B.** HCT116 cells were infected with lentiviral MYC shRNA or Luciferase (Luc) shRNA and treated with 30μM 2′CMeA. Cells were harvested and stained with Annexin V and 7-AAD. A representative flow cytometry analysis (left) and quantification of percent Annexin V positive cells (middle) are shown. Whole cell lysates were analyzed by Western blot for the indicated proteins (right). Error bars represent SEM, *n* = 3. *p < .01; n.s., not significant, Unpaired *t*-test. Casp-3, caspase-3; FL, full length; CL, cleaved; UT, untreated.

In humans, very little structural similarity exists between POLRMT and the nuclear RNA polymerase molecules. Instead, human POLRMT is related to the T7 family of prokaryotic enzymes [32-34]. Thus, drugs that target POLRMT might have little effect on the other human RNA polymerase enzymes. Structural analysis and *in vitro* enzymatic assays show that polymerization of RNA by POLRMT is inhibited by 2′-C-methyl-adenosine (2′CMeA) [35]. Empirical analysis confirmed that treatment of U2OS cells with 2′CMeA, which is converted efficiently to 2′-C-methyl-ATP once taken up by cells, results in a dose-dependent block in mitochondrial transcription without a measurable effect on nuclear transcription (Supplemental Figure S3A). This compound was therefore used to assess whether blocking the enzymatic activity of POLRMT *via* a small molecule strategy results in synthetic lethality with MYC activation, as was observed when POLRMT levels were reduced using shRNA. Treatment of U2OS MYC/ER cells with 2′CMeA had no significant effect on viability in the absence of MYC activation (Figure 4A). As shown previously, MYC activation also had no significant effect on viability (Figures 3B and 4A). However, when POLRMT inhibition by 2′CMeA was combined with MYC activation, synthetic lethality was observed in a dose-dependent manner (Figure 4A). Inhibition of POLRMT by 2′CMeA was also synthetically lethal with oncogenic levels of endogenous MYC (Figures 4B and S3B). HCT116 cells treated with 2′CMeA exhibited high levels of apoptosis when MYC was present, while cells depleted of MYC by shRNA reverted to a basal level of apoptosis. Coupled with the findings from POLRMT shRNA studies, these data suggest that MYC activation programs the cell to become strictly dependent on an essential function of POLRMT for survival.

### Inhibition of mitochondrial translation is synthetically lethal with oncogenic MYC

**Figure 5:**
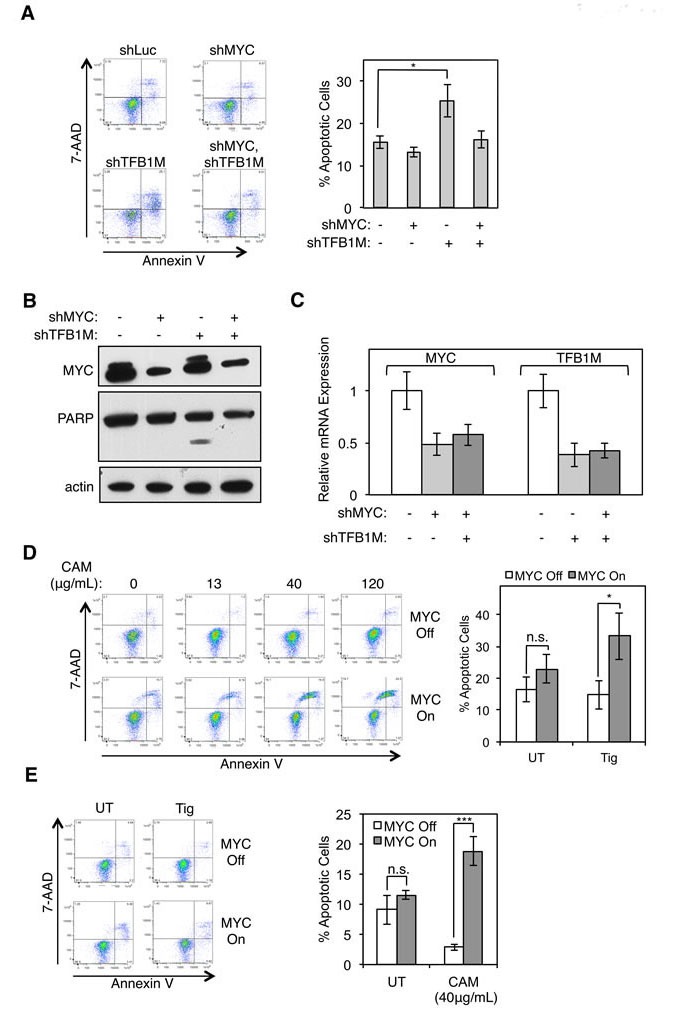
Inhibition of mitochondrial translation phenocopies synthetic lethality resulting from POLRMT inhibition **A.** HCT116 cells were infected with lentiviral MYC shRNA and/or TFB1M shRNA or Luciferase (Luc) shRNA. Five days post-infection cells were harvested and stained with Annexin V and 7-AAD. A representative flow cytometry analysis (left) and quantification of percent Annexin V positive cells (right) is shown. Error bars represent SEM, *n* = 5. *p < .05; n.s., not significant, Unpaired *t*-test **B.** Whole cell lysates of cells described in (A) were analyzed by Western blot for indicated proteins. **C.** Relative mRNA expression of cells described in (A) was measured by qRT-PCR. Error bars represent SD, *n* = 5. **D.** U2OS MYC/ER cells were treated with increasing concentrations of chloramphenicol, as indicated. MYC activity was induced *via* treatment with 4-OHT (MYC On). Three days post MYC-activation cells were harvested and stained with Annexin V and 7-AAD (left). Quantification of percent Annexin V positive cells at 40μg/mL dose is shown (right). Error bars represent SEM, *n* = 8. ***p < .0001; n.s., not significant, Unpaired *t*-test **E.** U2OS MYC/ER cells were treated with 10μM tigecycline (Tig) and MYC activity was induced *via* treatment with 4-OHT (MYC On). Three days post MYC-activation cells were harvested and stained with Annexin V and 7-AAD. A representative flow cytometry analysis (left) and quantification of percent Annexin V positive staining cells (right) is shown. Error bars represent SEM, *n* = 7. *p < .05; n.s., not significant, Unpaired *t*-test. UT, untreated.

In order to identify the essential function provided by POLRMT to MYC overexpressing cells, we explored the pathway of mitochondrial gene expression at multiple points of regulation. As discussed above, POLRMT is the rate-limiting enzyme of mitochondrial transcription and is also responsible for regulation of mtDNA replication [7, 26]. More recently, POLRMT was discovered to play a transcription-independent role *via* its interaction with a mitochondrial ribosomal RNA methyltransferase TFB1M [9]. The interaction between TFB1M and POLRMT is necessary to regulate mitochondrial ribosome assembly, and disruption of POLRMT expression leads to changes in steady-state levels of mitochondrial-encoded proteins. Thus, POLRMT is a critical regulator of mitochondrial translation, in addition to its roles in mitochondrial transcription and mtDNA replication. To functionally dissect the dependence of MYC-overexpressing cells on elevated mitochondrial gene expression, we inhibited mitochondrial translation in cells with oncogenic levels of MYC. Consistent with results using POLRMT shRNA, synthetic lethality was phenocopied when the POLRMT interaction partner TFB1M was depleted from HCT116 cells expressing high MYC levels (Figure 5A-5C), but there is a lack of significant apoptosis under conditions where MYC is depleted. Thus, blocking mitochondrial gene expression at the level of translation triggers the rapid death of MYC overexpressing cells. Supporting this, the Amati group recently found depletion of other components of the mitochondrial translation machinery to be synthetically lethal with oncogenic MYC, and we have also confirmed these results (Supplemental Figure S4) [36].

Several classes of antibiotics target bacterial ribosomes and prevent protein synthesis [37]. The bacterial translation machinery shares homology with the mitochondrial translation machinery in humans and therefore antibiotics targeting bacterial ribosomes also effect human mitochondrial ribosomes and mitochondrial protein synthesis [38]. Fortuitously, several FDA-approved antibiotics work *via* this mechanism. Supporting this as a potential therapeutic strategy in human cancer, recent studies have shown that targeting mitochondrial translation (and transcription) using antibiotics induced apoptosis of acute myeloid leukemia (AML) cells [39, 40]. However, no link to MYC levels was explored in the AML cells and the mechanism responsible for the sensitivity of cancer cell lines to blocking mitochondrial translation was not determined. In order to gain mechanistic insight into the apoptosis we have observed, we explored the effect of antibiotics that block mitochondrial translation in our MYC-inducible system. Upon MYC activation in U2OS MYC/ER cells, apoptosis was induced in response to chloramphenicol in a dose dependent manner (Figure 5D). Similarly, U2OS MYC/ER cells are sensitive to tigecycline, only in the presence of activated MYC (Figure 5E). Collectively, these results demonstrate that MYC-overexpressing cells are dependent on proper expression of mitochondrial encoded proteins for cell survival, a dependency that can be exploited using antibiotics to selectively induce the death of tumor cells expressing oncogenic levels of MYC.

### Tigecycline effectively reduces tumor burden *in vivo*

**Figure 6:**
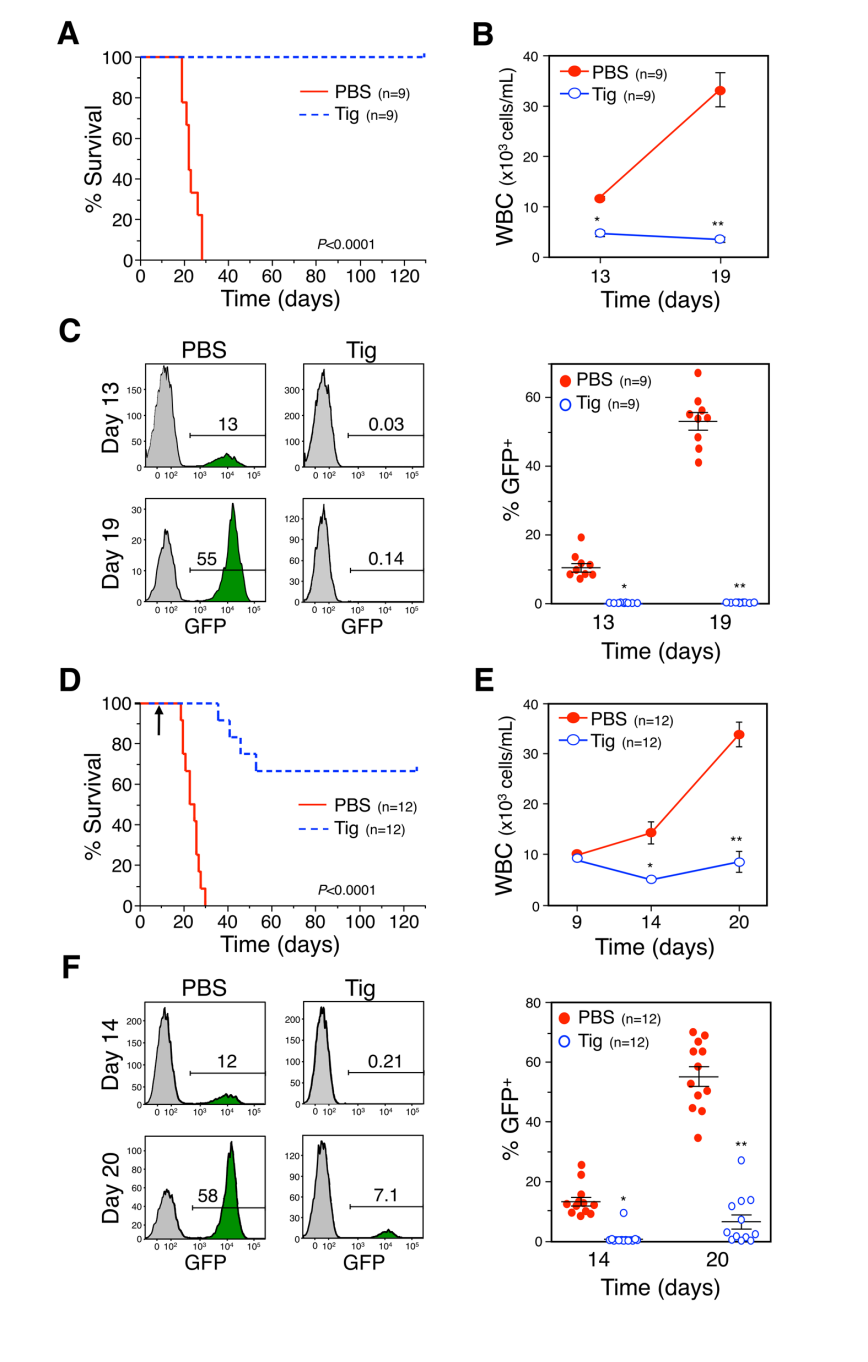
Tigecycline inhibits B cell lymphoma, *in vivo*, prolonging survival **A.** and **D.** Kaplan-Meier survival curves of C57Bl/6 mice injected intravenously with GFP-expressing Eμ-*myc* lymphoma cells and administered tigecycline (Tig) or PBS (vehicle control) starting the day of injection (A, *p* < 0.0001, log-rank test) or once the lymphoma had established on day nine indicated by an arrow (D, *p* < 0.0001, log-rank test). **B.** and **E.** For the mice in A and D, respectively, white blood cell counts (WBC) and GFP positive cells (lymphoma) in the blood, as measured by flow cytometry (**C** and **F**, respectively), were determined on the days indicated. The number (n) of mice in each group is indicated. B, *P = 4.43×10^−6^, **P = 1.57×10^−5^; C, *P = 1.58×10^−5^; **P = 1.57×10^−8^; E, *P = 7.23×10^−4^, **P = 9.42×10^−6^; F, *P = 4.77×10^−7^; **P = 4.02×10^−9^; *t*-tests.

Results showing that the FDA-approved drugs chloramphenicol and tigecycline can be used to target MYC-driven cancer cells *in vitro* prompted an examination of the effect of this treatment *in vivo*. Tigecycline is in wide clinical use [41] and has recently completed a Phase I clinical trial for the treatment of AML (clinicaltrials.gov ID: NCT01332786), based on the pre-clinical data mentioned above [39, 40]. To define the role of MYC in this phenomenon, an *in vivo* model of MYC-driven lymphomas was used to test the efficacy of tigecycline on preventing tumor progression. Mice were inoculated with GFP-expressing Eμ-*myc* lymphoma cells and treated with tigecycline or vehicle control (Figure 6). The transplantable lymphoma line derives from a primary tumor generated in mice which express the Eμ-MYC transgene which mimics the translocation of MYC to the *IgH* locus that is diagnostic of Burkitt's lymphoma [31, 42]. Mice inoculated intravenously with lymphoma cells derived from these primary tumors rapidly develop lymphomas and typically succumb to the disease within 30 days [43, 44]. However, treatment of these mice with tigecycline resulted in a dramatic reduction of the tumor burden, measured by white blood cell count and GFP-expressing cell count, and a subsequent improvement in overall survival. The experimental design included two arms of the study to explore therapeutic as well as preventative treatments. Mice were treated with tigecycline either before (Figure 6A-6C) or after (Figure 6D-6F) the lymphomas established, as measured by white blood cell count. Remarkably, mice treated before tumor development displayed 100% survival rate for 120 days after lymphoma cells were introduced, at which time the study was terminated (Figure 6A). Mice treated with tigecycline simultaneous with tumor cell inoculation retained a constant and normal number of white blood cells, whereas control mice displayed a steady increase in white blood cell count, reflecting lymphoma progression (Figure 6B). Consistent with this, GFP staining showed that control mice displayed a high percentage (approximately 60%) of lymphoma cells, while mice treated with tigecycline displayed less than 1% GFP-positive cells in blood samples at 19 days post-inoculation (Figures 6C, S5A and S5B). These results demonstrate that tigecycline treatment is effective at preventing MYC-driven lymphoma tumor engraftment and increasing survival *in vivo*. We also explored the therapeutic benefit of tigecycline treatment after lymphoma establishment. Notably, mice treated with tigecycline after lymphomas were established had approximately 70% survival rate with little increase in white blood cell count or GFP-positive cells (Figure 6D-6F, S5A and S5B). We presume that the effects of tigecycline on tumor cell survival are cell autonomous within the lymphoma cells, but there is a possibility the host stroma or immune system contribute to the effect of the drug. However, based on results found by the Amati group, tigecycline blocks the growth of MYC-derived lymphomas *in vivo*, even in the absence of an immune system (D'Andrea et al 2016, accompanying paper).

The marked success of tigecycline treatment *in vivo* highlights the potential for using this drug clinically to treat patients with MYC-driven tumors. One possible constraint of this treatment would be that MYC-dependent apoptosis relies on functional p53 in some cases. It was therefore worthwhile to determine whether apoptosis induced by simultaneous activation of MYC and blockade of mitochondrial gene expression requires an intact p53 pathway. To assess this potential dependency, isogenic HCT116 cells were utilized which are either wild-type or null for p53 [45]. In these cells, which express high endogenous MYC levels, depletion of POLRMT using two distinct shRNAs resulted in robust induction of apoptosis in the presence of p53, as measured by caspase-3 cleavage (Supplemental Figure S5C). In cells lacking p53 expression, depletion of POLRMT also led to an increase in caspase-3 cleavage compared to control cells (Supplemental Figure S5C). Though the levels of apoptosis were slightly diminished compared to p53 wild type cells, it is clear that POLRMT depletion remains synthetically lethal with oncogenic MYC regardless of p53 status. *In vivo* support for the p53 independence of these effects was provided by the fact that the Eμ-MYC lymphoma cells used in Figure 6 lack functional p53 [46], yet remain tigecycline sensitive.

### Tight coordination of the nuclear- and mitochondrial-encoded components of the mitochondrial program driven by MYC is critical for tumor cell survival

The results thus far support the hypothesis that induction of mitochondrial gene expression is required for the survival of MYC-overexpressing cells. In order to define the biochemical defect that triggers the apoptosis of cells expressing elevated MYC and depressed mitochondrial gene expression, several hypotheses were explored. One of these hypotheses relates to the role of MYC in regulating pyrimidine biosynthesis [47]. An essential step in this pathway involves the mitochondrial localized dihydrooratate dehydrogenase enzyme (DHODH), whose catalytic activity is dependent on ubiquinone as an electron acceptor. Dysfunctional electron transport chain (ETC) activity prevents ubiquinone production, and thereby impedes DHODH and pyrimidine biosynthesis. Rho0 cells, which lack mtDNA and therefore have defects in DHODH activity and pyrimidine biosynthesis, can survive if provided with exogenous uridine [48, 49]. To assess whether defective DHODH/pyrimidine biosynthesis could explain the synthetic lethality between MYC activation and POLRMT inhibition, U2OS MYC/ER cells were treated with uridine (and pyruvate). Pyruvate is necessary to maintain the cellular redox balance, which is altered in cells lacking mitochondrial oxidative phosphorylation due to an accumulation of NADH. Restoration of a normal redox state is achieved during the reaction converting pyruvate to lactate, while NADH is oxidized to NAD+ [49]. Cells supplemented with exogenous uridine displayed comparable levels of apoptosis to non-supplemented cells under synthetic lethal conditions (Supplemental Figures S6A and S6B), suggesting that a defect in pyrimidine biosynthesis is an unlikely trigger for apoptosis. As an alternative method to rescue this pathway, the yeast form of DHODH was expressed in these cells. The yeast DHODH enzyme is cytoplasmic rather than mitochondrial and does not require ubiquinone as an electron acceptor [50, 51]. Expression of this plasmid circumvents the requirement for ETC function in pyrimidine biosynthesis. As with the uridine/pyruvate supplementation study, expression of yeast DHODH did not reverse the synthetic lethality (Supplemental Figure S6D).

Another biological pathway that links MYC to mitochondria is one-carbon metabolism [52-54]. The serine hydroxymethyltransferase SHMT2 is a mitochondrial localized protein and a direct MYC target [52, 55]. In a screen for functional complementation, SHMT2 could provide partial rescue of the proliferation defect of MYC null cells [52]. We were prompted to explore the possibility that a defect in one-carbon metabolism, due to loss of mitochondrial SHMT2 function, could induce apoptosis in the presence of oncogenic MYC. However, depletion of SHMT2 in the presence of oncogenic MYC in U2OS MYC/ER cells had little effect on cell viability (Supplemental Figure S6E). These data suggest that MYC-overexpressing cells are not dependent on mitochondrial one-carbon metabolism regulated by SHMT2 for survival.

**Figure 7:**
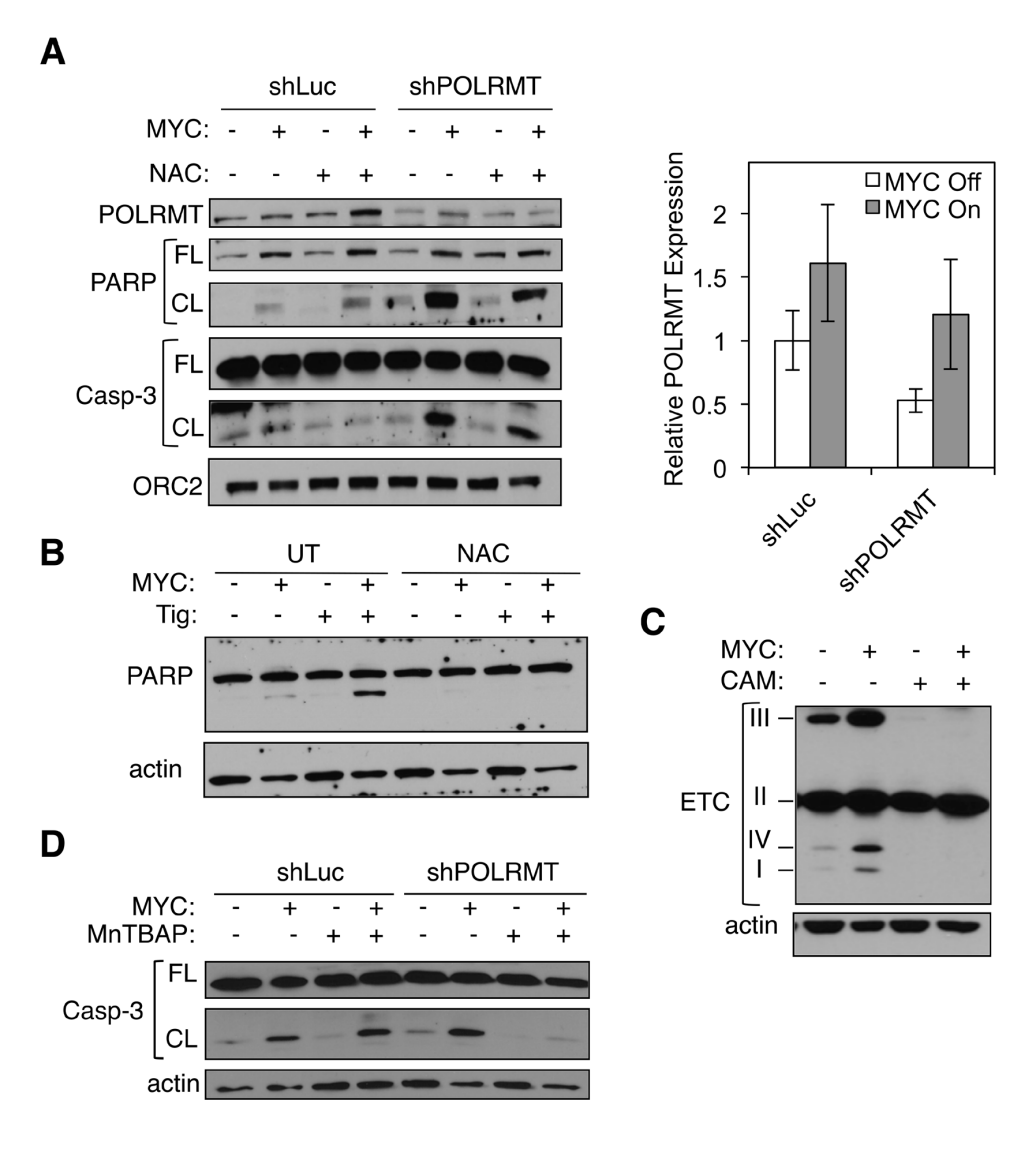
Mitochondrial gene expression protects MYC-overexpressing cells from toxic levels of ROS **A.** U2OS MYC/ER cells were infected with lentiviral POLRMT shRNA or Luciferase (Luc) shRNA and treated with NAC. MYC activity was induced *via* 4-OHT treatment (MYC On). Three days post-treatment cells were harvested and whole cell lysates were analyzed by Western blot for the indicated proteins (left). Relative mRNA expression was measured by qRT-PCR (right). Error bars represent SD, *n* = 3. **B.** U2OS MYC/ER cells were treated with tigecycline (Tig) and/or NAC. MYC activity was induced *via* 4-OHT treatment. Three days post-treatment cells were harvested and whole cell lysates were analyzed by Western blot for the indicated proteins. **C.** U2OS MYC/ER cells were treated with chloramphenicol (CAM). MYC activity was induced *via* 4-OHT treatment. Whole cell lysates were analyzed by Western blot using an antibody cocktail for subunits of electron transport chain (ETC) complexes, as indicated (I-IV). **D.** U2OS MYC/ER cells were infected with lentiviral POLRMT shRNA or Luciferase (Luc) shRNA and treated with MnTBAP. MYC activity was induced *via* 4-OHT treatment. Three days post-treatment cells were harvested. Whole cell lysates were analyzed by Western blot for the indicated proteins. Casp-3, caspase-3; FL, full length; CL, cleaved; UT, untreated.

The 13 genes encoded by the mitochondrial genome produce subunits of the ETC. Together with nuclear encoded subunits, these proteins form Complexes I, III, IV and V. As MYC is known to drive mitochondrial biogenesis and function, the nuclear encoded subunits of the ETC are overexpressed in the presence of oncogenic MYC [11]. It is possible that the combinatorial effect of increasing production of nuclear encoded subunits (due to high MYC), and simultaneous defects in production of mitochondrial encoded subunits (e.g. when POLRMT is depleted), causes an imbalance in the stoichiometry of subunits within individual ETC complexes. Defects in proper ETC activity cause release of electrons, thereby increasing cellular levels of reactive oxygen species (ROS), which are potent inducers of apoptosis [56-58]. In order to test the hypothesis that the synthetic lethality is triggered by high cellular ROS levels, we treated cells with ROS scavengers. U2OS MYC/ER cells were depleted of POLRMT and treated with 4-OHT to induce MYC activity, resulting in the synthetic lethal phenotype. Upon treatment with the ROS scavenger N-Acetyl Cysteine (NAC), synthetic lethality was partially reversed (Figure 7A). Similarly, induction of synthetic lethality *via* treatment with tigecycline was also rescued with NAC (Figure 7B). These results support the hypothesis that MYC coordinately regulates nuclear- and mitochondrial-encoded proteins to form a functional ETC, and that disturbing this balances triggers ROS production and apoptosis. Thus, cells with oncogenic MYC express high levels of nuclear-encoded ETC subunits in order to meet the metabolic demand of rapid growth and proliferation, but interfering with MYC's ability to coordinately regulate mitochondrial-encoded ETC subunits creates an imbalance in stoichiometry, leading to toxic cellular ROS levels and apoptosis.

To directly assess whether this imbalance exists, we analyzed the protein levels of ETC complex subunits that are labile when their resident complex is misassembled. As shown in Figure 7C, upon chloramphenicol treatment, cells lose stability of Complexes I, III and IV. Complex II is entirely nuclear-encoded, and is therefore unaffected by inhibition of mitochondrial translation. These observations are consistent with the synthetic lethality resulting from an imbalance in the stoichiometry of ETC complexes. Complex II is therefore overproduced, relative to the other complexes, presumably leading to release of electrons and increased levels of apoptosis [58]. To more distinctly define the source of ROS production, we performed synthetic lethality experiments using a ROS scavenger specific to mitochondrial-produced ROS. This reagent, MnTBAP, is a superoxide dismutase mimetic that targets the superoxide molecules produced by free radicals created during ETC reactions [59, 60]. As shown by caspase-3 cleavage, synthetic lethality was reversed upon treatment with MnTBAP (Figure 7D), confirming that the source of apoptosis is mitochondrial-produced ROS. Additionally, we directly measured levels of mitochondrial produced superoxide using the MitoSOX Red assay. As shown in Figure S6F, there is an increase in mitochondrial superoxide in cells under synthetic lethal conditions. Collectively, the data presented in this study indicate that MYC-overexpressing cells are reliant on elevated mitochondrial gene expression for proper ETC function and protection against mitochondrial-produced ROS. We have shown that this pathway can be targeted as a means to therapeutically treat MYC-driven cancers, both *in vitro* and *in vivo*. The therapeutic implications of this study are heightened by the existence of FDA-approved drugs that target this essential pathway.

## DISCUSSION

MYC overexpression is common to the majority of human cancers, yet attempts to directly target this oncogene as a therapeutic strategy have historically proven challenging. This led to the concept that targeting essential downstream nodes in the MYC pathway might be a more practical goal [61]. However, MYC regulates several thousand genes and only a few MYC targets are documented as required for the survival of MYC-overexpressing cells (i.e. essential downstream nodes) [13]. Thus, the identification of the essential downstream targets, from among the thousands of genes regulated by MYC, has also presented a challenge. Selective identification of these essential downstream targets was the rationale for the screen in which POLRMT was identified (4). The identification of POLRMT as an essential downstream effector of MYC subsequently led to the observation that MYC overexpression plays a central role in controlling the entire gene expression program at the mitochondrial genome. Furthermore, MYC-mediated increases in mitochondrial gene expression are critical to its ability to drive malignancy and blocking MYC's upregulation of mitochondrial gene expression triggers an acute apoptotic response. Notably, this apoptotic response can be triggered by inhibiting MYC's upregulation of mitochondrial gene expression at any of multiple steps, using either pharmacologic or genetic approaches. As discussed above, the Amati group recently identified the mitochondrial translation factor PTCD3 in an RNAi screen specifically designed to identify genes whose inhibition results in synthetic lethality with MYC overexpression (D'Andrea et al 2016, accompanying paper). Considering the distinct methodologies and cellular platforms utilized across the two studies, the convergent discovery of an essential requirement for enhanced mitochondrial gene expression adds support to the view that this newly identified link is of broad importance for MYC function.

The discovery that MYC controls the full mitochondrial transcription program via its direct regulation of the nuclear gene encoding the mitochondrial RNA polymerase also completes the scenario which began to emerge when MYC was documented to play a direct role in regulating all three RNA polymerase molecules that transcribe the nuclear genome (reviewed in [62]).

As mentioned, our own screen was designed to exploit the current understanding of the structure/function relationship of MYC protein domains. For many decades the highly conserved but functionally enigmatic MbII domain was known to be essential for MYC's ability to drive malignancy [63, 64]. We demonstrated that this requirement for MbII relates in part to its role in mediating interactions between MYC and transcriptional coactivator complexes containing the TRRAP acetyltransferase partner [65, 66]. Supporting our premise that MYC target genes linked to essential MYC functions might require the transcriptional cofactors recruited by the similarly essential MbII domain, TRRAP itself was identified by the Grandori group in a recent RNAi screen for genes whose depletion results in synthetic lethality with MYC activation [67]. Also consistent with this central concept, the two previously characterized MYC targets identified in our MbII-based screen, MTA1 and BAG1, were subsequently shown to play essential roles in MYC function [5, 6].

Mechanistically, a number of distinct biochemical defects could provide the explanation for the synthetic lethality observed between MYC activation and inhibition of mitochondrial gene expression. For example, Rho0 cells have a defect in pyrimidine biosynthesis due to impairment of the mitochondrial-localized DHODH enzyme and can survive only if supplied with exogenous sources of pyrimidine [68]. However, exogenous pyrimidine did not rescue the synthetic lethality observed here (Supplemental Figures S6A-D), suggesting that defective pyrimidine biosynthesis/DHODH activity is not the primary cause of cell death. Similarly, experimental results have largely eliminated the possibility that MYC's role in one carbon metabolism is the point of vulnerability that explains the synthetic lethality (Supplemental Figure S6E). Instead, data support a model in which MYC plays a critical role in enhancing mitochondrial function and biogenesis, partly via its known role in driving the nuclear gene expression program that encodes proteins imported into the mitochondria (10), and partly via the synchronous upregulation of the mitochondrial gene expression program, as reported here. In this model, blockade of the mitochondrial arm of this coordinated upregulation, whether by shPOLRMT, shTFB1M, 2′CMeA, chloramphenicol or tigecycline treatment, results in lethality caused by an imbalance in the mitochondrial- and nuclear-encoded components of the mitochondria (as evident in Figure 7C). Aberrant subunit stoichiometry results in misassembly of ETC complexes, a phenomenon known to cause electron leakage and subsequent mitochondrial ROS generation [58]. Consistent with this model, treatment with the mitochondria specific ROS scavenger MnTBAP blocks the synthetic lethal phenotype (Figure 7D). It is also noteworthy that the death observed upon POLRMT inhibition in MYC-driven cells is rapid, resembling more closely a checkpoint-like phenomenon than the cell death associated with the gradual attrition of mitochondria as cells proliferate.

Ultimately, these findings have several practical and conceptual implications for treatment strategies in oncology. The identification of enhanced mitochondrial gene expression as a targetable pathway could be rapidly translated into therapeutic approaches by utilizing FDA-approved compounds that work by mechanisms similar to tigecycline. As mentioned above, tigecycline itself has recently completed a Phase I clinical trial for treatment of AML (clinicaltrials.gov ID: NCT01332786). Based on TCGA data, MYC is elevated in only 9% of AML patients and neither the pre-clinical studies nor the AML clinical trial used MYC as a biomarker of tigecycline sensitivity [39]. The lack of homology between POLRMT and other mammalian RNA polymerase molecules also raises the possibility that targeting POLRMT with inhibitors mechanistically related to 2′CMeA could also prove efficacious and selective. The fact that all tested strategies for inhibiting mitochondrial gene expression have effects only when MYC is simultaneously expressed at oncogenic levels, provides optimism that a therapeutic window might exist between lethal effects of these treatments on tumor cells and toxicity in non-malignant cells. This principle appears broadly applicable since the synthetic lethality was observed in cells of many distinct lineages where oncogenic MYC levels were achieved by a variety of means. Also providing optimism for these findings having broad implications are the data showing that the synthetic lethality can be observed even in tumor cells lacking functional p53.

Recent efforts in the design of direct and indirect MYC inhibitors aim to decrease MYC expression/function as a therapeutic strategy [69-72]. In contrast, the findings reported here suggest that exploiting the potent apoptotic activity provided by MYC overexpression for a therapeutic advantage might be more advantageous. High expression (i.e. oncogenic) levels of MYC are required for apoptosis [73, 74], offering a unique opportunity to specifically target cancer cells. The approach of exploiting oncogene overexpression, rather than inhibiting oncogenic signaling pathways, offers a distinct perspective on the design of cancer therapies.

## MATERIALS AND METHODS

### Cell culture

Cell lines were obtained from ATCC. All cells, with the exception of Raji and IMR90 cells, were maintained in Dulbecco's modified Eagle's medium (DMEM) (Corning) supplemented with 10% fetal bovine serum (FBS) (Gemini). Raji cells were maintained in RPMI 1640 (Corning) supplemented with 10% FBS and 1% L-glutamine (Corning). IMR90 cells were maintained in Eagle's minimum essential medium (EMEM) (Corning) supplemented with 2mM L-glutamine, 1% nonessential amino acids (Corning) and 10% FBS.

The MYC/ER fusion protein was activated by adding 4-hydroxytamoxifen (4-OHT) (Sigma Aldrich) at a final concentration of 30nM. JQ1 was used as previously described [75]. The 2′C-methyladenosine (2′CMeA) was a generous gift of Dr. Joy Feng and Gilead Sciences, Inc and was added to cell media at concentrations ranging from 3-30μM. The antibiotics chloramphenicol and tigecycline were used at concentrations ranging from 13-120μg/mL and10μM, respectively (Sigma Aldrich). The ROS scavenger N-acetylcysteine (NAC) was used at a concentration of 5mM (Sigma Aldrich). The mitochondrial-specific ROS scavenger MnTBAP was used at a concentration of 100nM (MED Millipore). Exogenous uridine and pyruvate were used at concentrations of 50μg/mL and 100μg/mL, respectively (Sigma Aldrich).

**Table:** 

Target	TRCN	Sequence
MYC	0000039642	CCGGCCTGAGACAGATCAGCAACAACTCG AGTTGTTGCTGATCTGTCTCAGGTTTTTG
POLRMT-1	0000053114	CCGGCGGTGGATGTACCCATGCTTTCTCG AGAAAGCATGGGTACATCCACCGTTTTTG
POLRMT-2	0000053113	CCGGGCAGAACCACTACAGGAAGTACTCG AGTACTTCCTGTAGTGGTTCTGCTTTTTG
PTCD3	0000145035	CCGGGCTGATATCAAATCTGCGTATCTCG AGATACGCAGATTTGATATCAGCTTTTTTG
SHMT2	0000034807	CCGGGCTCCAGGATTTCAAATCCTTCTCG AGAAGGATTTGAAATCCTGGAGCTTTTTG
TFB1M	0000021041	CCGGCCACGATTCGAGAAATCATTACTCG AGTAATGATTTCTCGAATCGTGGTTTTT

**Table:** 

Human Gene	Forward Primer	Reverse Primer
12S	AAAACTGCTCGCCAGAACACTAC	GCACCCCCAGGTCCTTT
ATP6	TCATTCAACCAATAGCCCTGG	GCCTGCAGTAATGTTAGCGGT
CAD	TCAAAAGGTGTCCAGTGGGC	GACAATTCCCAGCCACATGC
CytB	CGCATGATGAAACTTCGGCT	ATTTGGAGGATCAGGCAGGC
HBO1	TCATGATGAGTCACCGCCTC	TCAGAAGAAGGCGCATTTCC
MYC	GGACGACGAGACCTTCATCAA	GCCGCTCCACATACAGTCCT
ND2	GGTTGCTTGCGTGAGGAAAT	AACCCTCGTTCCACAGAAGCT
ND4	CCTAAAGCCCATGTCGAAGC	TGCGGCAAGTACTATTGACCC
PCAF	ACCCTAACCCCTCACCCACT	CAATTATTTGCTGCAGGTCGG
POLRMT	CGGGTCCCCGAGAACATC	GGGAGGCCTCTCACTATCTCGTA
SHMT2	CAAGACTGGCCGGGAGATC	GGGAACACGGCAAAGTTGAT
SMYD2	TCATAGCTGTTGCCCCAATG	TCTGCCAGGGTCCCTTTGTA
TFB1M	TGCTTGCCGCGTATCATG	CGGAGGGAGACGGCAAGT
POLRMT-1 (ChIP)	GCTTTCTCTCCTTGCAGCCTC	GCTGGAAGACACAGCACGTG
POLRMT-2 (ChIP)	GGAGTCTACTTCCGGCTGGG	GTGGTTTCATGGTCGACG
POLRMT-3 (ChIP)	TGCCTTGAGCATTTCCTTCC	CCAGACAGATTCACGAGCCC
POLRMT-4 (ChIP)	CCGCGATGTGTGGTTAGATCT	GATTCTGCTGGAACGACCTCC

### Plasmid expression and lentiviral production

MYC siRNA was obtained from Dharmacon. GFP siRNA was used as a negative control. Cells were transfected with 200 nM siRNA per well in 6-well plates using DharmaFECT 2 (Dharmacon). Cells were harvested and RNA and protein extracted 72 hours after transfection. The Plv-based lentiviral expression of human POLRMT was obtained from Dr. Chumakov. Lentiviral short hairpin RNA (shRNA) plasmids corresponding to MYC, POLRMT, TFB1M, SHMT2 and control luciferase shRNAs were obtained from TRC (Sigma). See below table for shRNA sequences. All sequences are from listed 5′-3′. Lentiviral packaging plasmids (pCMV-R8.2 and pCMV-VSV-G) were cotransfected with vectors into 293T cells. Viral supernatants were collected, filtered, and added directly to target cells in the presence of 8μg/ml polybrene (Sigma Aldrich). Cells were selected with puromycin (Sigma Aldrich) for 24 hours. The yDHODH retroviral vector was constructed in the packaging vector MIGR1. Retroviral packaging plasmid Ψ2 was cotransfected with yDHODH or empty vector into U2OS MYC/ER cells in the presence of 8μg/mL polybrene. Transfected cells were isolated by fluorescence-activated cell sorting (FACS) for the GFP marker expressed from the retroviral vector, 2-3 days post-transfection.

### Quantitative RT-PCR and chromatin immunoprecipitation

Quantitative RT-PCR (qRT-PCR) was performed by real-time analysis using the Step One Plus detection system (Applied Biosystems) and SYBR GREEN PCR Master Mix kit (Applied Biosystems). RNA was isolated using the Trizol method (Invitrogen) and cDNA was then generated using the High-Capacity cDNA Reverse Transcription Kit (Applied Biosystems).

ChIP was performed as described previously [5], using chromatin from the primary human fibroblast strain 2091. See below table for primer sequences. All sequences are listed from 5′-3′.

### Western blotting

To generate whole cell extracts, cells were lysed with E1A lysis buffer (20mM NaH2PO4, 150mM NaCl, 0.5% IGEPAL, 5mM EDTA, 50mM NaF, 30mM Sodium pyrophosphate, 10% Glycerol). The following antibodies were used to detect protein expression by Western blot, MYC, actin, p53, H2B (Santa Cruz), POLRMT, Total OXPHOS Cocktail (Abcam), Caspase-3 (Cell Signaling), PARP (Biovision), γH2A.X (Millipore), and ORC2 (BD Bioscience). Western blotting of mitochondrial isolates for the mitochondrial proteins POLRMT, TFAM, HSP60 and porin has been described previously [7].

### mtDNA quantitation

DNA was collected from whole cell lysates. qPCR was performed for genomic sequences of the mitochondrial encoded genes cytochrome c oxidase subunit 1 (COX1), NADH dehydrogenase subunit 2 (ND2), ATP synthase 6 (ATP6), NADH dehydrogenase subunit 4 (ND4), 12S rRNA, and the nuclear gene peroxisome proliferative activated receptor gamma coactivator- related 1 (PPRC1). Absolute COX1, ND2, ATP6, ND4 and 12S DNA copies were normalized to nuclear genome content, as represented by PPRC1 signals. As a control, a nuclear genome sequence at 3p24 was amplified.

### Proliferation assay

2×10^5^ U2OS MYC/ER cells were plated in complete medium. After attachment of cells to tissue culture dishes (approximately 24 hours), cells were treated with 4-OHT as described above. Cells were harvested and counted by hemocytometer after 3 and 5 days of treatment.

### Apoptosis assays

To quantitate apoptotic cell death, cells were collected by trypsinization, washed with phosphate-buffered saline and stained using the Annexin V PE-7AAD apoptosis detection kit (BD Pharmingen) as described previously [76]. Fluorescence was detected by flow cytometry (BD LSRII) and analyzed using FlowJo software (TreeStar). 10,000 total events were collected and then subsequently analyzed for the percentage of Annexin V-PE positive cells.

### ROS assay

To quantitate levels of reactive oxygen species, cells were collected by trypsinization, washed with phosphate-buffered saline, and counted. 200,000 cells were stained with MitoSOX Red Mitochondrial Superoxide Indicator (Thermo Fisher). Fluorescence was detected by flow cytometry (BD LSRII) and analyzed using FlowJo software (TreeStar). 10,000 total events were collected.

### Mice

Female bitransgenic MMTV-rtTA;TetO-MYC mice [16, 77] were administered 2 mg/mL doxycycline in drinking water beginning at 6 weeks of age to induce c-MYC expression in mammary tissue and mice were palpated weekly to identify mammary tumors. Mammary tumors were harvested from mice on doxycycline, as well as from tumor-bearing mice following 96 hr of doxycycline withdrawal to induce c-MYC down-regulation. Tumor tissue was snap frozen and RNA was extracted and purified as described above.

C57Bl/6 mice (6-11 weeks old) were injected (intravenous, tail vein) with 2×10^6^ Eμ-myc lymphoma cells expressing GFP. Tigecycline (100 mg/kg) or PBS (vehicle control) was injected (intraperitoneal) twice daily for 3 days followed by once daily for 11 days. Treatment commenced the day of lymphoma injection for one cohort of mice (*n* = 9 per group) or in a second cohort (*n* = 12 per group), once the lymphoma had established (day 9) as determined by elevated (~9-10×10^3^ cells/ml) white blood cell counts. Blood was collected at intervals to measure white blood cell counts (WBC) using a FORCYTE Analyzer (Oxford Science) and the percentage of GFP positive lymphoma cells using flow cytometry (see below). All mice were carefully monitored and sacrificed at humane endpoints as the lymphoma progressed. All surviving mice were sacrificed on day 129 or 126 following lymphoma injection for the first or second cohort, respectively. Kaplan-Meier analyses were performed and log-rank tests determined statistical significance for survival (GraphPad Prism).

Whole blood (30 ml) was drawn from each mouse at intervals. Following hypotonic lysis of red blood cells, GFP positive cells (lymphoma) were measured by flow cytometry (BD FACSCanto II) and analyzed using FlowJo software (TreeStar). Lymph nodes were removed from surviving mice at the end of the study. Single cell suspensions were prepared from the lymph nodes and subjected to flow cytometry.

### Oncomine microarray datasets

Gene expression of MYC, POLRMT, LDHA and BAG1 was analyzed using microarray gene expression datasets deposited in the Oncomine Cancer Profiling Database (www.oncomine.org). Each gene was queried individually and Primary Filters were set to Analysis Type: Cancer *vs*. Normal Analysis. The Basso Lymphoma dataset comparing Burkitt's Lymphoma *vs*. Normal was used to display gene expression values. Additional datasets used to compare MYC and POLRMT expression include Skryzpczak Colorectal 2, Sabates-Bellver Colon, and Zhan Myeloma.

### UCSC genome browser

MYC binding sites at the POLRMT locus were identified using the UCSC Genome Browser Assembly GRCh37/hg19 with a display of the track for Transcription Factor ChIP-seq Uniform Peaks from ENCODE [23, 24].

## SUPPLEMENTARY MATERIALS FIGURES


