# Supplementary material for: Multi-focal control of mitochondrial gene expression by oncogenic MYC provides potential therapeutic targets in cancer
Source: Oncotarget. 2016 Aug 31;7(45):72395–414. doi: 10.18632/oncotarget.11718 (PMC5340124; doi:10.18632/oncotarget.11718)
Supplement: Supplementary file 1 [file oncotarget-07-72395-s001.pdf]

Multi-focal control of mitochondrial gene expression by oncogenic MYC provides potential therapeutic targets in cancer

Supplementary Material

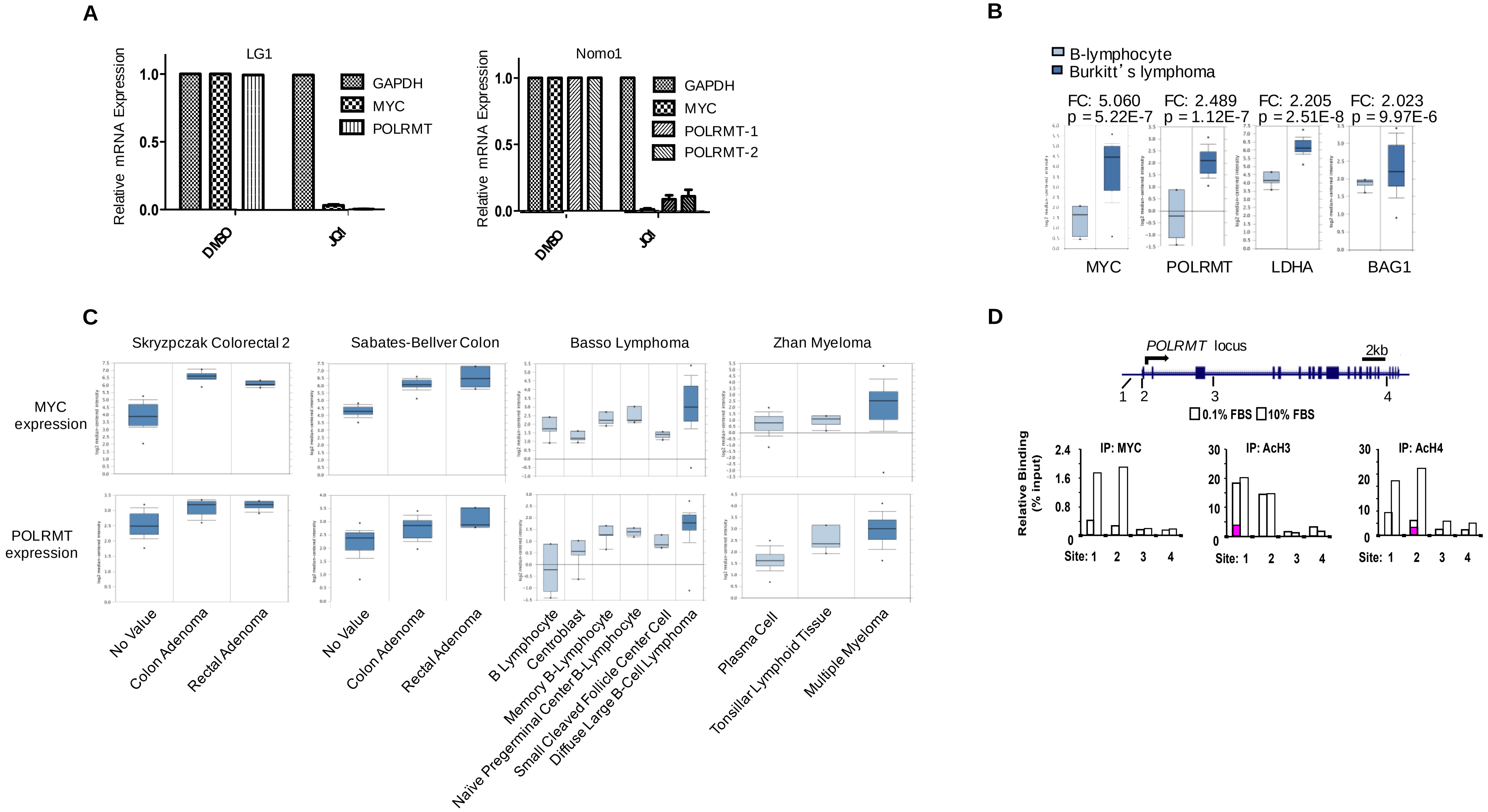
A
LG1
Relative mRNA Expression
1.0
0.5
0.0
GAPDH
MYC
POLRMT
DMSO
JQ1
Nomo1
GAPDH
MYC
POLRMT-1
POLRMT-2
B
B-lymphocyte
Burkitt's lymphoma
FC: 5.060
p = 5.22E-7
FC: 2.489
p = 1.12E-7
FC: 2.205
p = 2.51E-8
FC: 2.023
p = 9.97E-6
MYC
POLRMT
LDHA
BAG1
C
Skryzpczak Colorectal 2
Sabates-Bellver Colon
Basso Lymphoma
Zhan Myeloma
MYC expression
POLRMT expression
No Value
Colon Adenoma
Rectal Adenoma
B Lymphocyte
Centroblast
Memory B-Lymphocyte
Naïve Pregerminal Center B-Lymphocyte
Small Cleaved Follicle Center Cell
Diffuse Large B-Cell Lymphoma
Plasma Cell
Tonsillar Lymphoid Tissue
Multiple Myeloma
D
POLRMT locus
2kb
1
2
3
4
0.1% FBS
10% FBS
Relative Binding (% input)
IP: MYC
IP: AcH3
IP: AcH4
Site: 1
2
3
4

Figure S1, Related to Figure 1. (A) LG1 (left) and Nomo1 (right) cells were treated with 500nM JQ1 for 24 hours to deplete MYC expression. Relative mRNA expression was quantified by qRT-PCR. **(B)** Basso Lymphoma dataset analyzed using Oncomine (www.oncomine.org) shows differential expression comparing Burkitt's lymphoma samples to B-lymphocytes. P values and Fold Change (FC) are shown. **(C)** Analysis of additional datasets using Oncomine show POLRMT and MYC expression in a variety of cancer types. The name of the dataset is displayed above each set of graphs and the cell type is labeled below. **(D)** MYC was induced in growth factor-deprived primary human fibroblasts by serum stimulation for two hours. ChIP was performed to detect occupancy of the selected regions (top) of the *POLRMT* locus by MYC, or by acetyl-histones H3 or H4 (bottom). Rabbit IgG was used in control ChIP experiments.

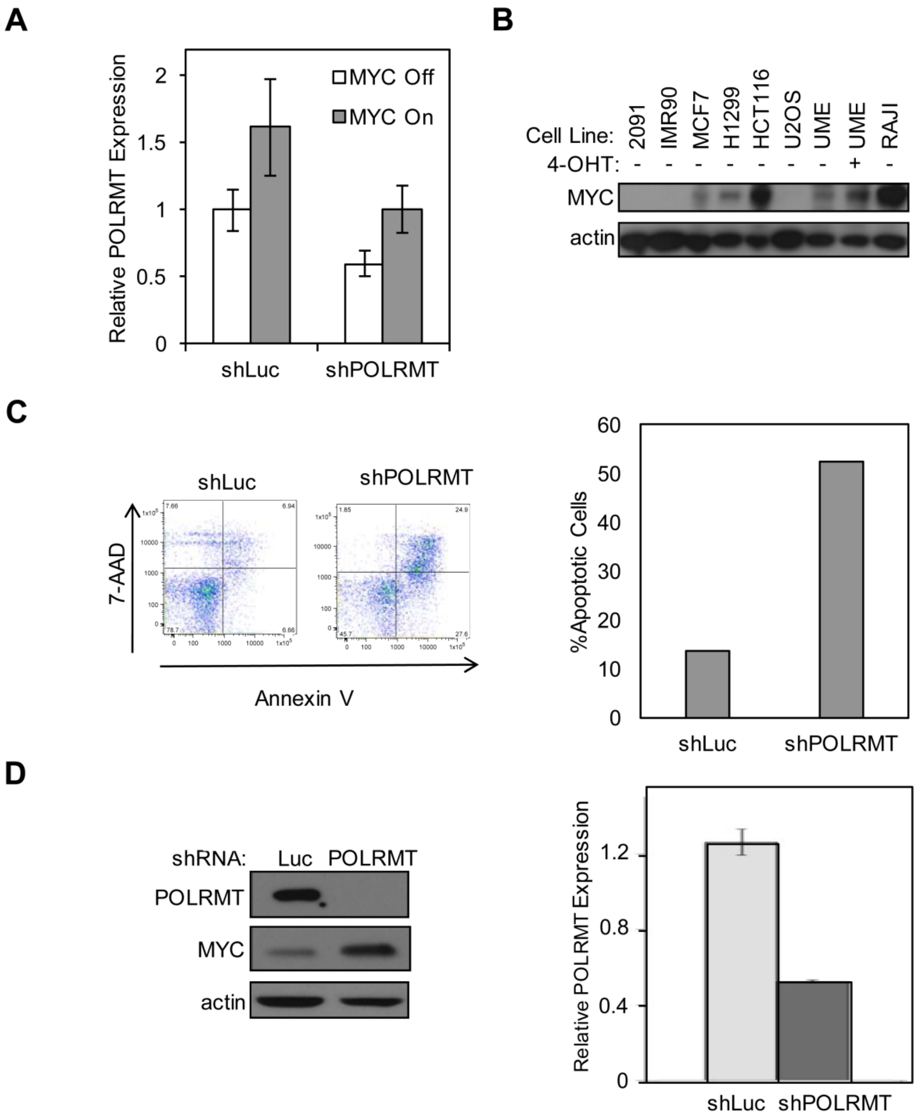

Figure S2, Related to Figure 3. (A) Relative POLRMT mRNA expression of cells described in Figure 3B was measured by qRT-PCR. Error bars represent SD. **(B)** MYC expression across multiple cell lines shown by Western blot of whole cell lysates. UME, U2OS MYC/ER. **(C)** Raji cells were infected with lentiviral POLRMT shRNA or Luciferase (Luc) shRNA. Cells were harvested and stained with Annexin V and 7-AAD. Shown are flow cytometry analysis (left) and

quantification of percent Annexin V positive cells (right). **(D)** Cells described in (S2C) were analyzed by Western blot of whole cell lysates for the indicated proteins (left) and qRT-PCR (right). Error bars represent SD.

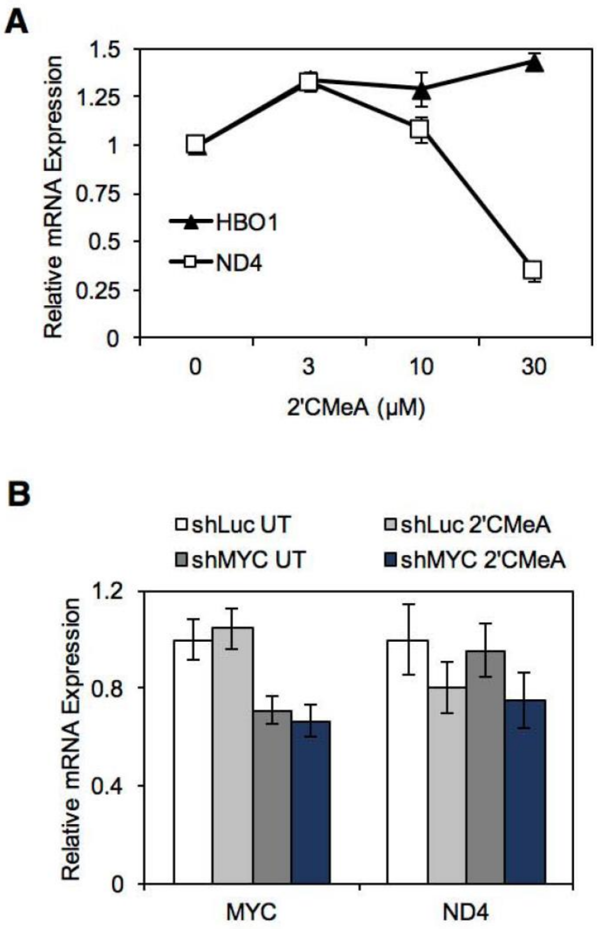

Figure S3, Related to Figure 4. (A) U2OS MYC/ER cells were treated with increasing concentrations of 2’CMeA, as indicated. Expression of nuclear transcript HBO1 and mitochondrial transcript ND4 were analyzed by qRT-PCR. Error bars represent SD. **(B)** Relative mRNA expression of cells described in Figure 4B was measured by qRT-PCR. Error bars represent SD.

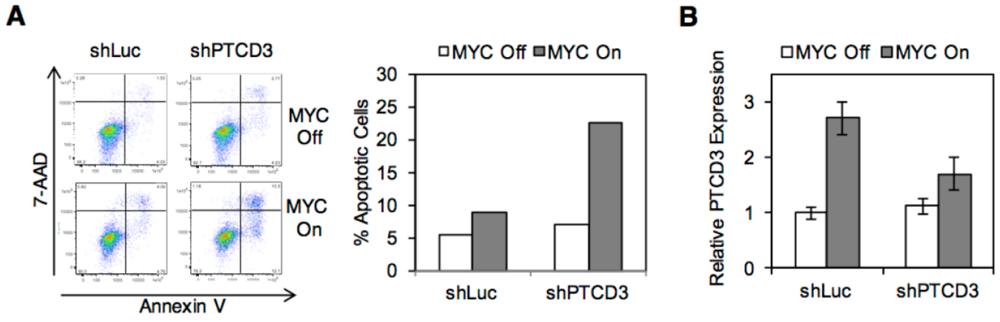

Figure S4, Related to Figure 5. (A) U2OS MYC/ER cells were infected with lentiviral PTCD3 shRNA or Luciferase (Luc) shRNA as a control. Five days post-infection cells were treated with 4-OHT (MYC On). Three days post MYC-activation cells were harvested and stained with Annexin V and 7-AAD (left). Quantification of percent Annexin V positive cells is shown (right). **(B)** Relative mRNA expression of cells described in (A) was measured by qRT-PCR. Error bars represent SD.

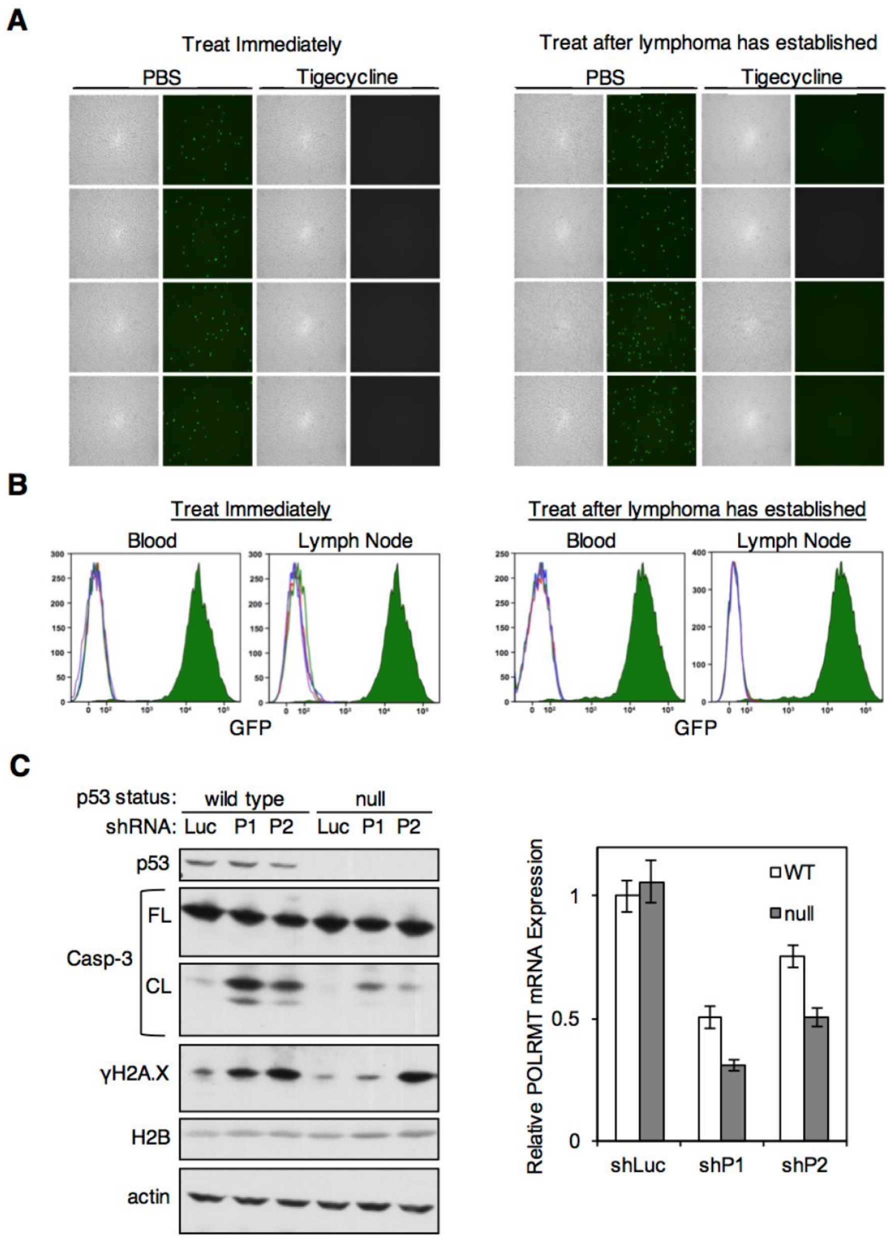

Figure S5, Related to Figure 6. (A) Left: Eμ-*myc* lymphoma cells expressing GFP were injected intravenously into C57Bl/6 mice who were administered tigecycline or PBS (vehicle control) the same day (treat immediately) for 14 days. Microscopic images (white light left and GFP fluorescence right) of whole blood with GFP positive lymphoma cells in four representative mice of each group 19

days post lymphoma injection. Right: Eμ-*myc* lymphoma cells expressing GFP were injected intravenously into C57Bl/6 mice. Tigecycline or PBS (vehicle control) was administered for 14 days after the lymphoma established (day nine). Microscopic images (white light left and GFP fluorescence right) of whole blood with GFP positive lymphoma cells in four representative mice of each group 20 days post lymphoma injection. **(B)** Histograms of GFP-positivity in cells from the blood and lymph nodes of surviving mice from Figure 6A and 6D was measured by flow cytometry. Representative data from four mice (each curve different color) from each cohort compared to control (green peak). The control is cultured GFP-expressing Eμ-*myc* lymphoma cells that were injected into the mice initially. **(C)** Isogenic HCT116 cells either wild type (WT) or null for p53 were infected with one of two lentiviral POLRMT shRNA plasmids (P1 and P2) or Luciferase shRNA (Luc). Cells were harvested and whole cell lysates were analyzed by Western blot for the indicated proteins (left). POLRMT expression was measured by qRT-PCR (right). Error bars represent SD. Casp-3, caspase-3; FL, full length; CL, cleaved.

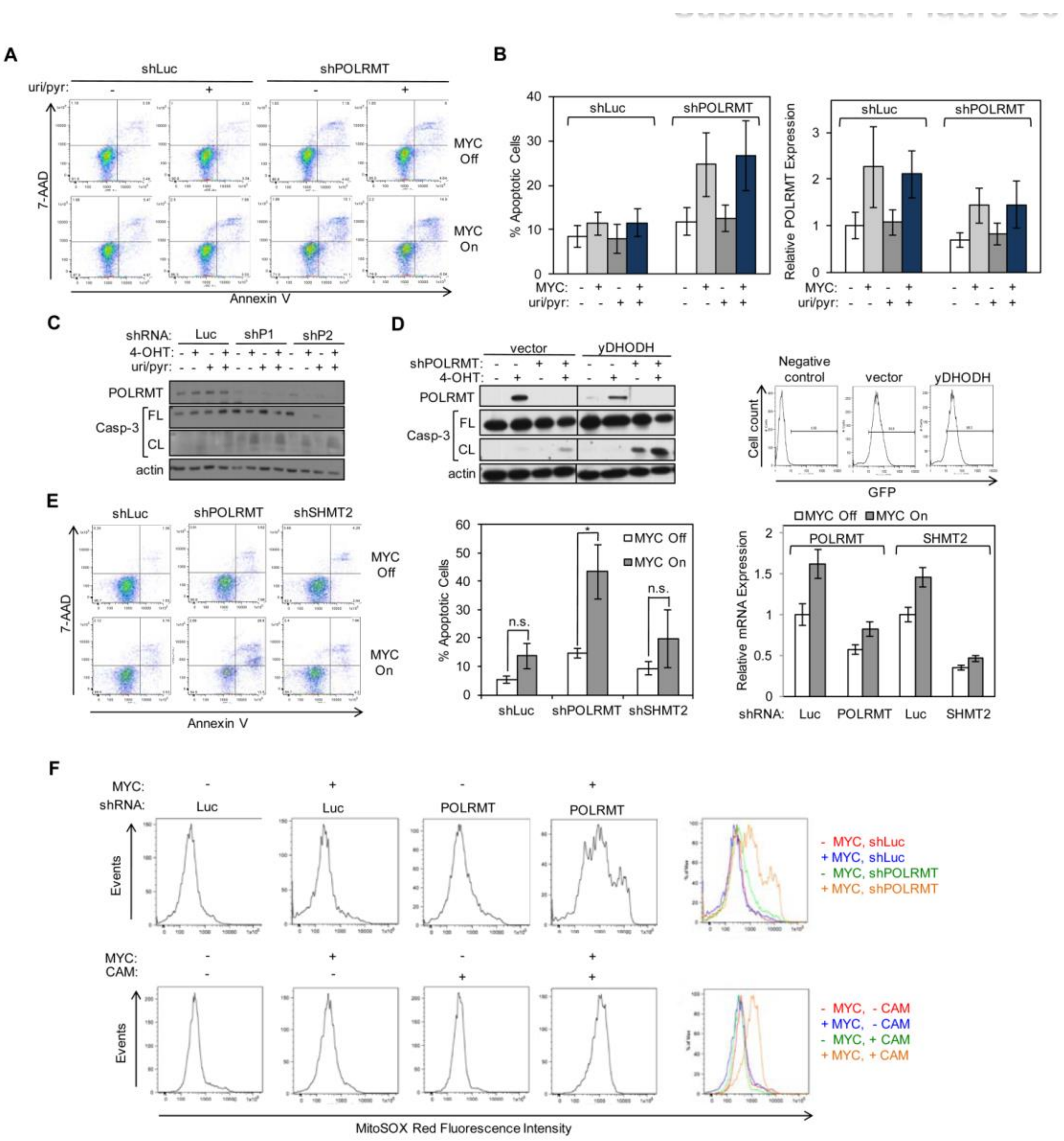

Figure S6, Related to Figure 7. (A) U2OS MYC/ER cells were infected with lentiviral POLRMT shRNA or Luciferase (Luc) shRNA. Cells were treated with 4-OHT and/or uridine and pyruvate (uri/pyr). Three days post-treatment cells were harvested and stained for Annexin V and 7-AAD. A representative flow cytometry analysis is shown. **(B)** Quantification of the percent of Annexin V positive staining cells (left) and POLRMT mRNA expression measured by qRT-PCR (right) for

cells described in (A). Error bars represent SEM (left) and SD (right). **(C)** U2OS MYC/ER cells were infected with two distinct lentiviral POLRMT shRNA plasmids (shP1 and shP2) or Luciferase shRNA. Cells were treated as in (A). Whole cell lysates were analyzed by Western blot for the indicated proteins. **(D)** U2OS MYC/ER cells were stably transfected with yeast DHODH (yDHODH) or empty vector and sorted for GFP. Cells were infected with lentiviral POLRMT shRNA or Luciferase shRNA. MYC activity was induced via treatment with 4-OHT. Three days post MYC-activation cells were harvested and whole cell lysates were analyzed for the indicated proteins (left). GFP expression is shown (right). **(E)** U2OS MYC/ER cells were infected with lentiviral POLRMT shRNA, SHMT2 shRNA, or Luciferase (Luc) shRNA. MYC activity was induced via treatment with 4-OHT (MYC On). Three days post MYC activation cells were harvested and stained with Annexin V and 7-AAD. A representative flow cytometry analysis (left) and quantification of Annexin V positive cells (middle) is shown. POLRMT and SHMT2 expression were measured by qRT-PCR (left). Error bars represent SEM (middle), n=3, *p<.05; and SD (right), n=2. Casp-3, caspas-3; FL, full length; CL, cleaved. **(F)** U2OS MYC/ER cells were infected with shRNA to deplete POLRMT (top) or treated with chloramphenicol (bottom) and treated with 4-OHT to induce MYC activity. Three days post MYC activation cells were harvested and stained with MitoSOX Red. Fluorescence was measured by flow cytometry.
